# Structural plasticity of the membrane-bound protein degradation assembly supports bacterial adaptation to stress

**DOI:** 10.1016/j.celrep.2026.117231

**Published:** 2026-04-10

**Authors:** Naseer Iqbal, Sandro Keller, Alireza Ghanbarpour

**Affiliations:** 1 Department of Biochemistry and Molecular Biophysics, Washington University School of Medicine, St. Louis, MO, USA; 2 Biophysics, Institute of Molecular Biosciences (IMB), NAWI Graz, University of Graz, 8010 Graz, Austria; 3 Field of Excellence BioHealth, University of Graz, 8010 Graz, Austria; 4 BioTechMed-Graz, 8010 Graz, Austria; 5 Lead contact

## Abstract

Protein degradation by ATPases associated with diverse cellular activities) (AAA+) proteases is essential for bacterial adaptation to stress. The membrane-bound protease FtsH forms an inner-membrane complex with the SPFH (stomatin, prohibitin, flotillin, and HflK/C) (SPFH) proteins HflK and HflC that promotes recovery from aminoglycoside antibiotics. Although open and closed HflK/C conformations have been described, their functional relevance has remained unclear. Here, we engineer a disulfide-crosslinked HflK/C variant to stabilize the closed state and determine its structure by high-resolution cryo-electron microscopy (cryo-EM). Cells expressing this variant, or an HflK/C mutant that disrupts FtsH binding, exhibit impaired growth under aminoglycoside stress, demonstrating that conformational dynamics and productive HflK/C-FtsH interactions are required for adaptation. Surprisingly, cryo-EM of the FtsH·HflK/C complex from tobramycin-treated cells reveals a distinct conformation with two openings that may facilitate substrate entry during proteotoxic stress. Together, these findings establish HflK/C conformational flexibility as a determinant of stress adaptation and provide a framework for understanding SPFH protein function.

## In brief

Iqbal et al. show that HflK/C conformational dynamics regulate bacterial adaptation to aminoglycoside stress. Using disulfide crosslinking to constrain the closed state, they demonstrate that stabilizing a closed HflK/C assembly impairs stress recovery and reveal a stress-induced conformation with dual openings that may facilitate FtsH-dependent membrane proteolysis.

## Graphical abstract

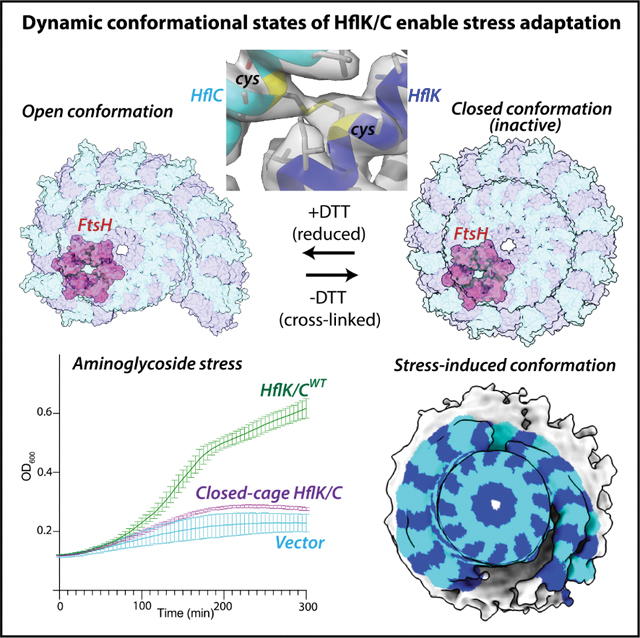

## INTRODUCTION

Dynamic protein degradation allows organisms, from bacteria to humans, to regulate their proteome in response to diverse cellular signals.^1^ In bacteria, this process is primarily mediated by five major ATPases associated with diverse cellular activities (AAA+) proteases, with FtsH being the only membrane-spanning member, which degrades both soluble and membrane proteins.^2,3^ Structurally, the FtsH monomer consists of a transmembrane helix, a periplasmic domain, a second transmembrane helix, and a cytoplasmic AAA+ module, followed by a zinc-dependent peptidase domain.^4,5^

The AAA+ domain recognizes substrates via a degron sequence and uses ATP hydrolysis to generate the mechanical force needed for substrate unfolding and translocation into the peptidase chamber for degradation.^6,7^ In *Escherichia coli*, FtsH is essential, as it plays a pivotal role in lipid metabolism by removing key enzymes involved in lipopolysaccharide (LPS) biosynthesis, including LpxC and KdtA.^8,9^ This degradation helps balance phospholipid and LPS synthesis, thereby regulating outer membrane permeability and ensuring proper cell-envelope biogenesis.^9^ Additionally, FtsH degrades unpartnered SecY and additional misfolded or damaged membrane proteins, helping maintain the integrity of protein translocation across the inner membrane.^10^ In eukaryotes, mitochondrial FtsH homologs are essential for protein quality control, with dysregulation linked to neurodegenerative and metabolic diseases.^11,12^

FtsH associates with the single-transmembrane proteins HflK and HflC to form a massive complex in the bacterial inner membrane.^13^ HflK and HflC belong to the stomatin, prohibitin, flotillin, and HflK/C (SPFH) protein family, which is conserved across bacteria and eukaryotes and is known to organize membrane microdomains and regulate membrane-associated processes.^14–16^ Although the HflK/C complex is dispensable under normal conditions, it becomes essential during stress, such as exposure to aminoglycoside antibiotics or oxidative conditions,^17^ which result in the accumulation of unfolded or damaged proteins that must be cleared in an FtsH- and HflK/C-dependent manner.^18^ However, the molecular mechanism by which HflK/C functions under aminoglycoside antibiotic stress or oxidative conditions is not completely understood, and only inhibitory roles have so far been associated with this complex.^18^ Deletion of *hflK* and *hflC* also impairs bacterial aerobic respiration,^19^ resembling mitochondrial defects caused by prohibitin inactivation in yeast and mammalian cells.^19,20^

Previous studies involving overproduction of the complex demonstrated that HflK/C forms a closed cage around four copies of FtsH.^21,22^ Based on this structure, the authors proposed a model in which the HflK/C cage inhibits FtsH-mediated proteolysis of membrane-protein substrates.^21,22^ Through the extraction of the endogenous complex using both detergent-based and detergent-free methods, we have previously revealed a markedly different arrangement: two hexameric FtsH proteases embedded within an open, asymmetric, nautilus-like HflK/C assembly when cells were grown under normal non-stressed conditions.^4^ Based on structural and proteomic analyses, we proposed that the HflK/C assembly modulates FtsH activity both positively and negatively.^4^

Herein, to elucidate how different conformations of the complex contribute to bacterial recovery from stress, we engineered a disulfide-crosslinked variant of HflK/C that stably adopts a closed conformation. Compared to the wild-type HflK/C (HflK/C^**WT**^), phenotypic assays revealed that the closed-conformation variant impaired bacterial growth during treatment with tobramycin, an aminoglycoside antibiotic that induces membrane-associated stress.^18^ Cryo-electron microscopy (cryo-EM) analysis of native complexes extracted from tobramycin-treated cells revealed a new conformation of the FtsH·HflK/C membrane complex, featuring two openings positioned adjacent to both FtsH hexamers. Together, our structural and phenotypic data support a model in which conformational flexibility of the HflK/C complex is essential for regulating FtsH activity and facilitating bacterial adaptation to antibiotic stress. These findings provide insight into SPFH protein conformational dynamics and add to the growing body of structural information in a field where many reported structures have captured closed conformations.^21–26^

## RESULTS

### Designing disulfide bond crosslinks between HflK and HflC to stabilize the closed conformation

To investigate the biological significance of the open, nautilus-like assembly and its opening in regulating membrane-protein degradation by FtsH, we engineered a disulfide bond crosslink between HflK and HflC to stabilize the closed HflK/C assembly.

**Figure 1.**
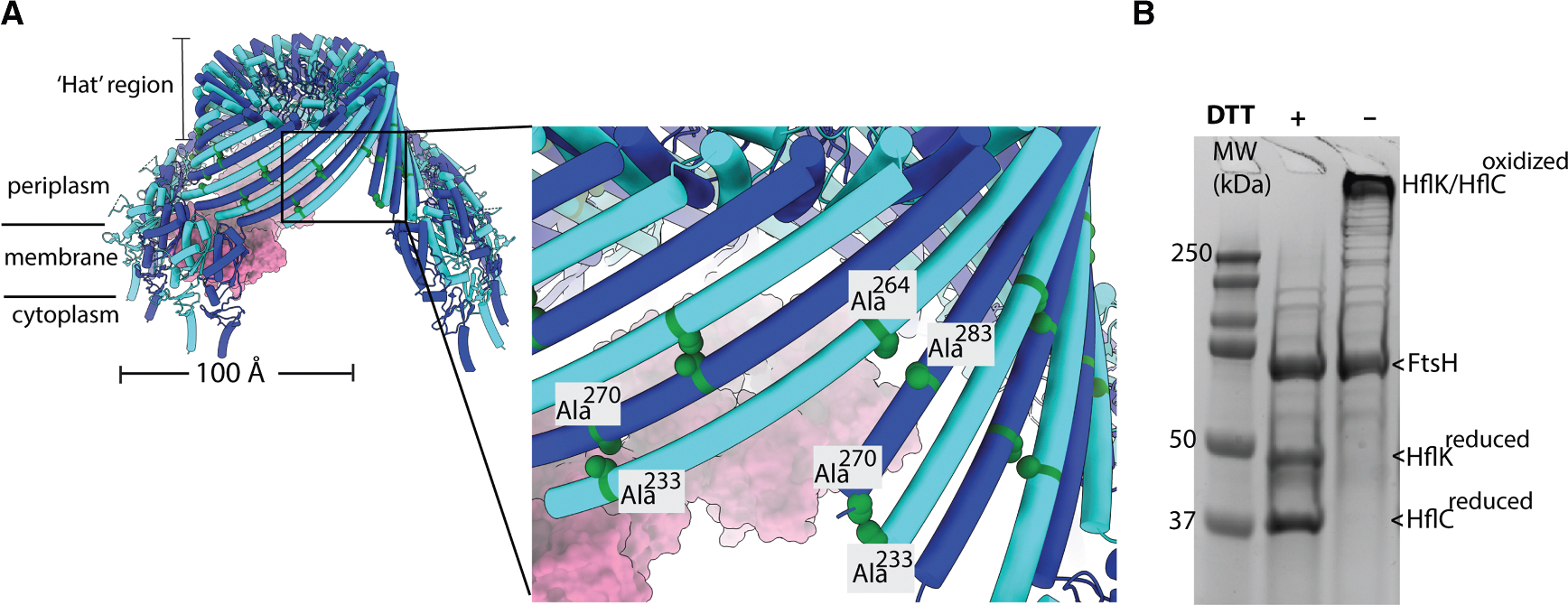
Designing disulfide bonds between HflK and HflC to stabilize the closed conformation of the HflK/C membrane assembly (A) Positions of cysteine substitutions in HflK (Ala^270^ and Ala^283^) and HflC (Ala^233^ and Ala^264^). The mutations are designed in the hat region of the structure and allow each HflK to be crosslinked to two neighboring HflC subunits, and vice versa. HflK and HflC are shown in blue and cyan, respectively, in the cartoon representation. (B) SDS-PAGE analysis of affinity-purified FtsH·HflK/C (right lane) under reducing and non-reducing conditions, with molecular weight standards in the left lane. Under reducing conditions, distinct bands corresponding to HflK and HflC are observed; under oxidizing conditions, these merge into a high-molecular-weight band that does not migrate through the gel, indicating disulfide bond formation under non-reducing conditions.

Guided by our previous structural data (Protein Data Bank [PDB]: 9CZ2), we introduced cysteine substitutions at alanine residues 270 and 283 in HflK and at residues 233 and 264 in HflC, with the goal of generating two disulfide bonds that connect each HflK to two adjacent HflC subunits, and vice versa (Figure 1A). These positions were selected for three reasons. First, the “hat” portion of the structure is relatively rigid, as it shows limited conformational variability across available structures.^4,21^This structural rigidity enhances the likelihood of successful disulfide bond formation, as the targeted residues are not subject to significant positional shifts (Figure 1A). Second, the opening observed in our previous structure (PDB: 9CZ2) originates from the coiled-coil domain of four HflK/C subunits (Figure 1A, close-up view). The last modeled residues in this flexible region still include the engineered cysteine mutations. Therefore, introducing disulfide bonds at these sites was expected to restrict conformational flexibility and stabilize the closed conformation. Third, because these residues are located in the periplasmic space, the oxidative environment naturally promotes disulfide bond formation.

### Validation of the disulfide-crosslinked HflK/C and its interaction with FtsH

We expressed the disulfide-crosslinked HflK/C (HflK/C^**SS**^) mutant using a low-copy-number plasmid under the control of a sodium-propionate-titratable promoter in *E. coli* BL21 cells lacking the endogenous *hflK/C* genes. Additionally, these cells harbored a chromosomally encoded FLAG tag at the C terminus of FtsH to facilitate complex pull-down.^4^ Following expression of HflK/C^**SS**^, the complex was affinity purified using M2-FLAG resin. SDS-PAGE analysis of the eluate under reducing conditions confirmed successful co-purification of FtsH along with two additional protein bands corresponding to the sizes of HflK and HflC (Figure 1B). Notably, under non-reducing conditions, the HflK and HflC bands merged into a single high-molecular-weight species that failed to migrate into the gel, consistent with the formation of a larger HflK/C assembly stabilized by disulfide bonds. The ratio of band intensities on SDS-PAGE for the HflK and HflC mutants relative to FtsH was comparable to that of HflK/C^**WT**^, indicating that disulfide bond formation did not significantly affect the interaction between FtsH and HflK/C.

### Cryo-EM structure of the closed HflK/C assembly

**Figure 2.**
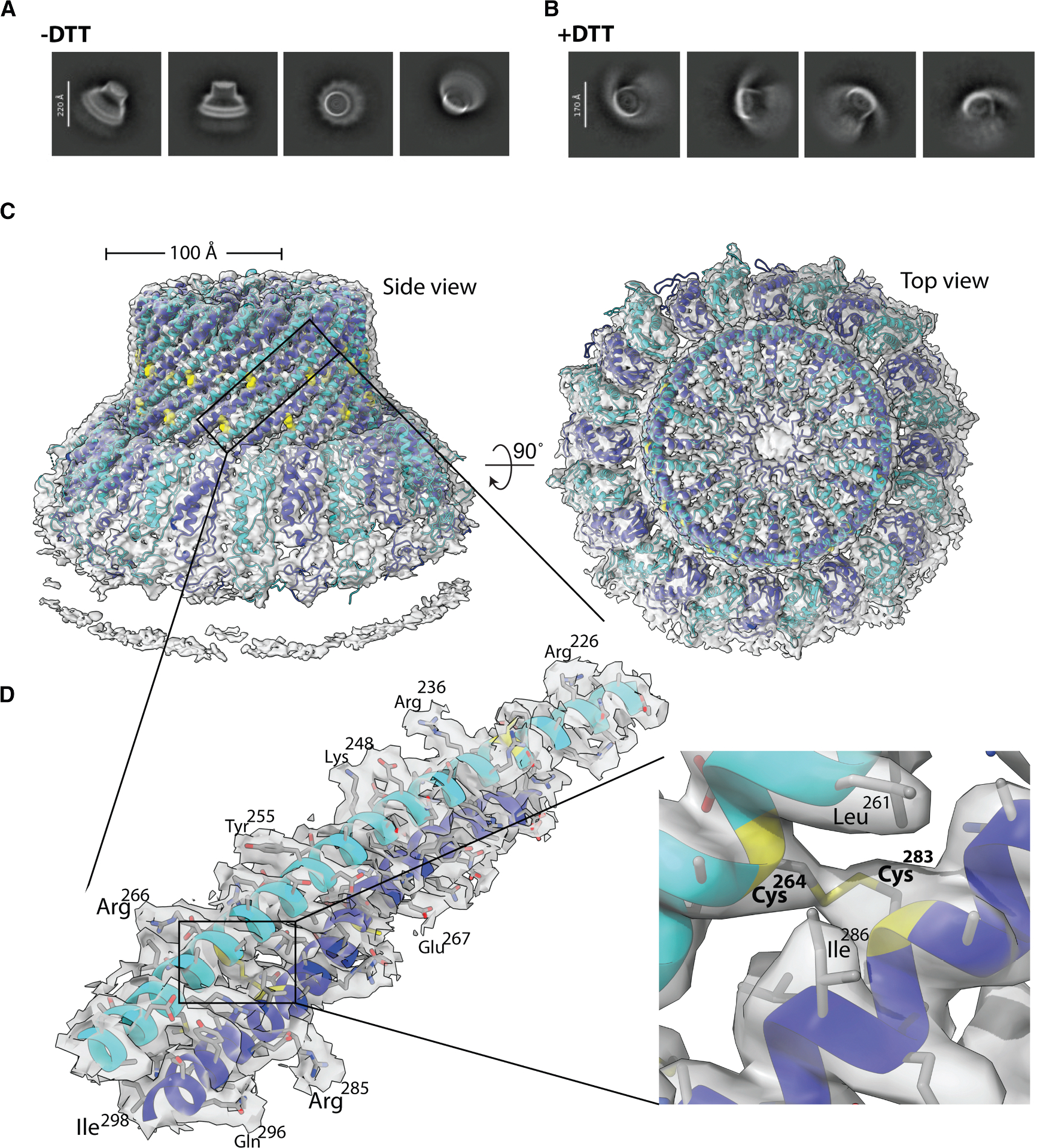
Cryo-EM structure of the DDM-solubilized, crosslinked FtsH·HflK/C^SS^ complex (A) 2D class averages of the HflK/C^SS^ (HflK/C^SS^) variant under non-reducing conditions show the closed conformation of the assembly. (B) Under reducing conditions, HflK/C^SS^ adopts an open, nautilus-like conformation. (C) Density map and cartoon models of HflK (blue) and HflC (cyan) viewed from the side and top, highlighting the closed HflK/C chamber. (D) Left: close-up view of the overlay between the atomic model and density map of the hat region of HflK (blue) and HflC (cyan), showing exemplary side-chain density. Right: an exemplary density for the disulfide bond between HflK and HflC.

**Figure 3.**
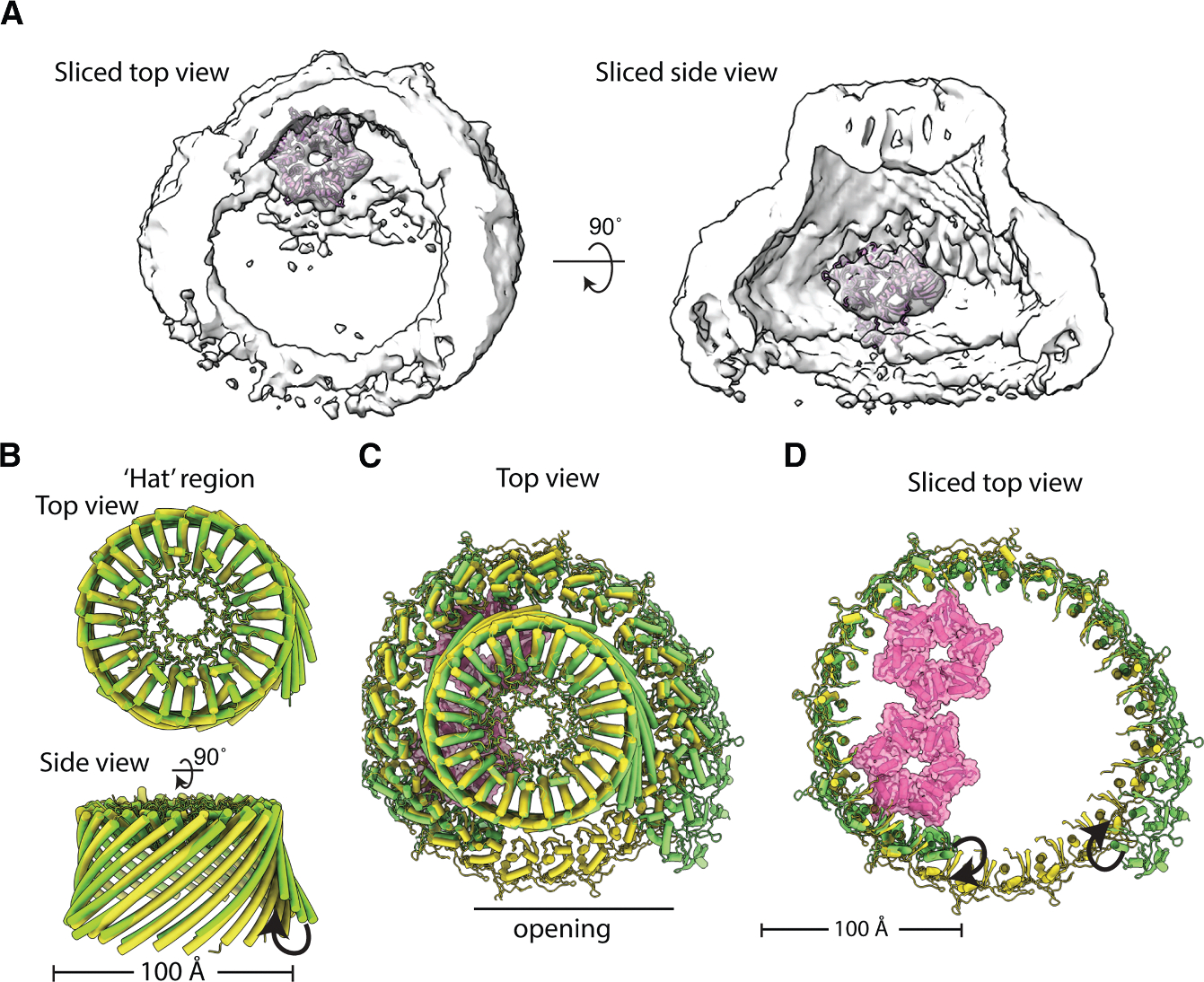
Comparison of the crosslinked HflK/C^SS^ closed conformation with the open structure (A) The sliced top and side view representations of the DDM-solubilized HflK/C^SS^ complex map obtained from heterogeneous refinement Figure S4), showing one FtsH residing inside the closed HflK/C assembly. (B) Overlay of the hat region from the crosslinked HflK/C^SS^ (yellow) and the open, nautilus-like structure (PDB: 9CZ2, green), shown in top and side views. (C) Top-view overlay of the full HflK/C^SS^ and the open, nautilus-like structure (PDB: 9CZ2) reveals that the overall arrangement is largely conserved; conformational differences are localized to the HflK and HflC subunits at the opening of the nautilus-like conformation (root-mean-square deviation [RMSD] = 1.7 Å). (D) Sliced top view of (C), highlighting that the positions of FtsH subunits (pink) from the nautilus-like conformation (PDB: 9CZ2) do not cause any steric clashes with the closed HflK/C^SS^ conformation. Arrows indicate the direction of the movement of the HflK and HflC subunits from the open to the closed structure.

To verify that crosslinking between HflK and HflC did not cause aberrant aggregation of the massive HflK/C complex, we prepared cryo-EM grids of the FtsH·HflK/C^**SS**^ complex solubilized in n-dodecyl β-D-maltoside (DDM) and glyco-diosgenin (GDN) (Figures 2 and S1–S3). Initial screening revealed 2D class averages consistent with a fully closed conformation of the HflK/C^**SS**^ cage under non-reducing conditions (Figure 2A). We also prepared another cryo-EM grid using the complex after incubation with DTT for 1 h at 30°C (Figure 2B). Screening of this grid revealed an open, nautilus-like assembly resembling HflK/C^**WT**^ (PDB: 9CZ2),^4^ indicating that the alanine-to-cysteine mutations *per se* did not disrupt the open, nautilus-like HflK/C conformation and that disulfide bond formation enforced the closed state. Following 3D reconstruction of the oxidized FtsH·HflK/C^**SS**^ complex, we determined the cryo-EM structure of HflK/C^**SS**^ at a global resolution of 2.9 Å in DDM using C_1_ symmetry (Figures 2 and S1–S3; Table 1). The density map resolved 24 copies of HflK/C, with the exception of the N and C termini of HflK, which are predicted to be unstructured,^4^ and the transmembrane regions of HflK/C and FtsH, which showed poor density (Figure 2; Table 1). Although the entire complex was pulled down via FtsH, FtsH itself was not seen in the final 3D reconstruction—most likely due to the high symmetry of the closed HflK/C^**SS**^ structure, where each of the 12 HflK subunits from different complexes could potentially interact with one or two FtsH hexamers, resulting in signal averaging that obscures the FtsH density. Supporting this notion, low-pass filtering the map to 20 Å to attenuate high-frequency noise revealed a density likely corresponding to the FtsH. To further resolve FtsH and confirm its presence within the assembly, we performed heterogeneity analyses using focused masks targeting the periplasmic region of FtsH, which allowed visualization of density corresponding to a FtsH periplasmic domain within the HflK/C assembly (Figures 3A, S4, and S5). The cytoplasmic AAA+ domain of FtsH is known to adopt multiple conformations depending on nucleotide state and is connected to the rest of the complex via a flexible linker^27^; this inherent flexibility likely limited its resolution in the final reconstruction, as previous high-resolution structures of FtsH or its homologs were often obtained using crosslinking, imposed symmetry, or mutations that slow ATPase activity.^22,28,29^ Because the reconstructions remained dominated by the rigid HflK/C scaffold, we additionally employed signal subtraction followed by local refinement to enhance the FtsH periplasmic signal. The resulting reconstruction of the periplasmic domain overlaid well with the previously published structure (PDB: 9CZ2) (Figure S5).

Comparison of the HflK/C^**SS**^ structure with two previously published structures (PDB: 9CZ2 and 7WI3)^4,22^ revealed that the hat portion of the assembly, where the disulfide bonds were introduced, was largely unaffected by crosslinking, except in the region where the opening originates in our earlier nautilus-like structure (Figures 3B–3D and S6). Moreover, the overall arrangement of HflK/C subunits that forms the complex, which we refer to as the curvature of the assembly, is similar between the closed conformation reported here and our previously determined open conformation (Figures 3B–3D), aside from subunits located at the opening of the HflK/C cage. Consistent with this, the closed conformation does not sterically clash with the positions of the two FtsH hexamers previously resolved in the open, nautilus-like structure. In contrast, a comparison between the two closed conformations, our current structure and a previously published one,^22^ revealed a notable difference in the overall curvature of the HflK/C assembly (Figure S6). Since the hat regions, where our disulfide bonds are located, were structurally similar, we suggest that the curvature observed in the earlier study (PDB: 7WI3) may have resulted from the use of the non-specific crosslinker glutaraldehyde during sample preparation and/or the imposition of C_4_ symmetry during 3D refinement.^22^

### Closed HflK/C conformation compromises bacterial stress response to aminoglycoside antibiotic stress

**Figure 4.**
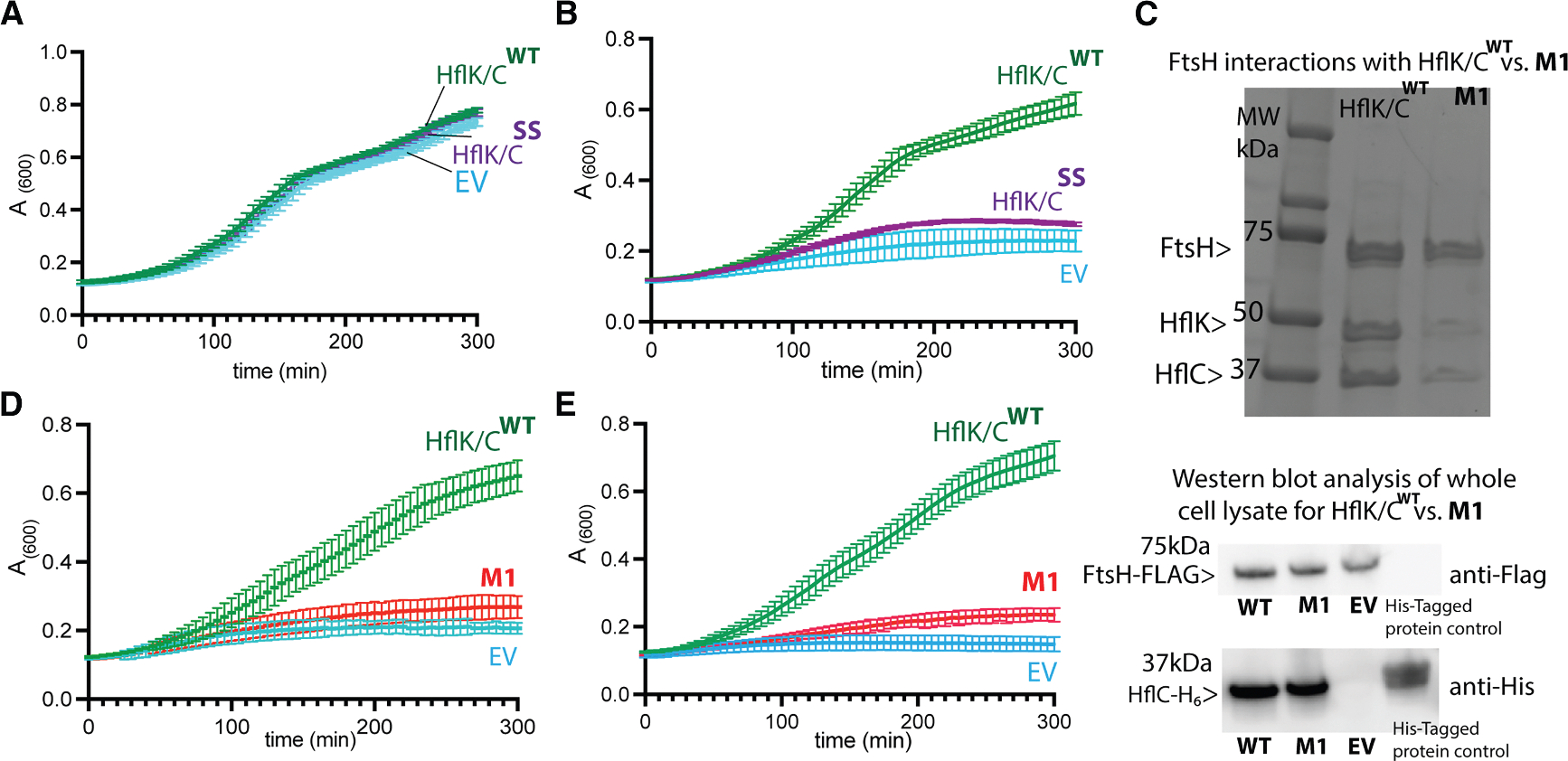
Effects of HflK/C variants on bacterial recovery under aminoglycoside stress All assays were performed using *E. coli* BL21 Δ*hflK/C* cells expressing various HflK/C variants from a pPro24 plasmid under the control of a sodium-propionate-inducible promoter at 37°C in LB medium containing 50 μg/mL kanamycin, 35 μg/mL chloramphenicol, and 1 mM sodium propionate unless otherwise noted. (A) Growth curves for cells expressing wild-type HflK/C (HflK/C^WT^), crosslinked HflK/C (HflK/C^SS^), and empty vector (EV). (B) Growth curves for cells expressing HflK/C^WT^, crosslinked HflK/C (HflK/C^SS^), and EV under tobramycin stress. (C) Top: pull-down of the HflK/C complex solubilized in DDM from *E. coli* BL21 *ΔhflK/C* cells with chromosomally encoded FLAG-tagged FtsH and either WT or M1 (R141A, E142A, and R185A) HflK, Bottom: western blot analysis of the whole-cell lysates shows a comparable expression of HflC between WT and M1 mutant. (D and E) Growth curves of tobramycin-stressed cells expressing: HflK/C^WT^, mutant M1 (HflK(R141A, E142A, R185A)/HflC), and EV under 1 mM sodium propionate (D) and 5 mM sodium propionate (E). Each growth assay was performed using a separate colony (*n* = 3 independent biological replicates), and data are presented as the mean ± 1 SD.

Having established a construct that exclusively favors the closed conformation, we next evaluated the impact of a closed HflK/C assembly on bacterial physiology under aminoglycoside stress. Although HflK/C is non-essential under normal conditions in *E. coli*, both FtsH and HflK/C are critical for recovery from aminoglycoside-induced stress, which compromises membrane integrity and leads to the accumulation of misfolded proteins requiring FtsH-mediated degradation.^17,18^ To confirm previous studies showing that both *ftsH* and *hflK/C* are upregulated under such stress,^18,30^ we performed RNA-seq analysis comparing tobramycin-treated and untreated *E. coli* BL21 cells (Figure S7). We observed upregulation of both *ftsH* and *hflK/C*, as well as known FtsH substrates such as *mgtA*, which is involved in regulating intracellular ionic strength^31,32^; *ibpA*, a small heat shock protein that buffers protein aggregation under heat or oxidative stress^33^; and other chaperones, including ClpB and endopeptidases, were also upregulated, suggesting an interplay between proteolysis and chaperone-mediated rescue (Figure S7; Table S1). To assess the functional relevance of the closed conformation of the HflK/C assembly, we performed plate-reader-based growth assays (Figures 4 and S8)^17^ in *BL21* Δ*hflKC* cells complemented with either HflK/C^**WT**^, the crosslinked variant (HflK/C^**SS**^), or an empty vector (EV), all expressed from the same low-copy-number plasmid used in our structural studies. In the absence of antibiotic stress, all strains grew similarly well at 37°C (Figure 4A), consistent with the non-essentiality of *hflKC* under standard growth conditions.^4,19^ However, under tobramycin stress, only cells expressing HflK/C^**WT**^ recovered, while those expressing the crosslinked variant (HflK/C^**SS**^) or the EV showed significantly impaired growth (Figure 4B). Given that we demonstrated that HflK/C^**SS**^ can adopt an open, nautilus-like conformation under reducing conditions (Figures 2A and 2B), we hypothesized that the addition of DTT might permit recovery from antibiotic stress by promoting transition to the open state through reduction of the engineered disulfide bonds. However, treatment with 1 mM DTT did not restore growth (Figure S9). This likely reflects that partial reduction of the engineered disulfide bonds is insufficient to rescue HflK/C function and that complete reduction of all 24 disulfide bonds is not achievable in the oxidative periplasmic environment without inducing substantial cellular toxicity (Figure S9).

### FtsH-HflK/C interactions are required for bacterial stress recovery

Previous studies have shown that FtsH associates with the HflK/C assembly exclusively through its interactions with HflK^13^ and that mutations in hydrophilic residues within the periplasmic domain of FtsH^22^ can significantly affect its interaction with the HflK/C complex. To investigate the functional role of this interaction in bacterial growth under antibiotic stress, we introduced mutations in HflK rather than in FtsH to avoid disrupting FtsH’s catalytic activity. Guided by our previous structures,^4^ we substituted three hydrophilic residues in HflK (Arg141, Glu142, and Arg185) with alanine to disrupt potential contacts with FtsH (**M1** mutant; Figure S10). The pull-down assay using FLAG-tagged FtsH recovered HflK and HflC in the **M1** background at levels lower than those in the WT (Figure 4C), suggesting that these residues are important for FtsH-HflK/C interactions. To rule out the possibility that reduced recovery was due to impaired expression or stability of the HflK/C complex, we appended a C-terminal His-tag to HflC in both **WT** and **M1** backgrounds. As previously reported,^34^ HflC is unstable in the absence of HflK and thus serves as a readout for HflK/C complex formation. Immunoblot analysis revealed similar levels of HflC in **WT** and **M1** strains, confirming that the **M1** mutations do not compromise HflK/C levels. Functionally, cells expressing the **M1** variant from a propionate-titratable promoter at both low (1 mM) and high (5 mM) sodium propionate concentrations showed impaired recovery under tobramycin stress compared to those expressing HflK/C^**WT**^ (Figures 4D, 4E, and S11), supporting a critical role for the FtsH-HflK/C interaction in promoting stress adaptation. Because the *in vivo* experiments described above were performed in the Δ*hflKC* BL21(DE3) strain used for our structural studies, which harbors additional mutations relative to *E. coli* K-12 strains, we next tested whether the observed phenotypes were strain specific. We therefore repeated the growth assays for both the HflK/C^**SS**^ construct and the **M1** mutant in a Δ*hflKC* MG1655 (K-12) background (Figure S12). Similar to the BL21(DE3) strain, neither stabilization of HflK/C in the closed conformation nor disruption of FtsH-HflK/C interactions by the **M1** mutation restored recovery under tobramycin stress in MG1655 (Figure S12).

### The crosslinked HflK/C and the M1 mutant retain scramblase activity similar to the WT

**Figure 5.**
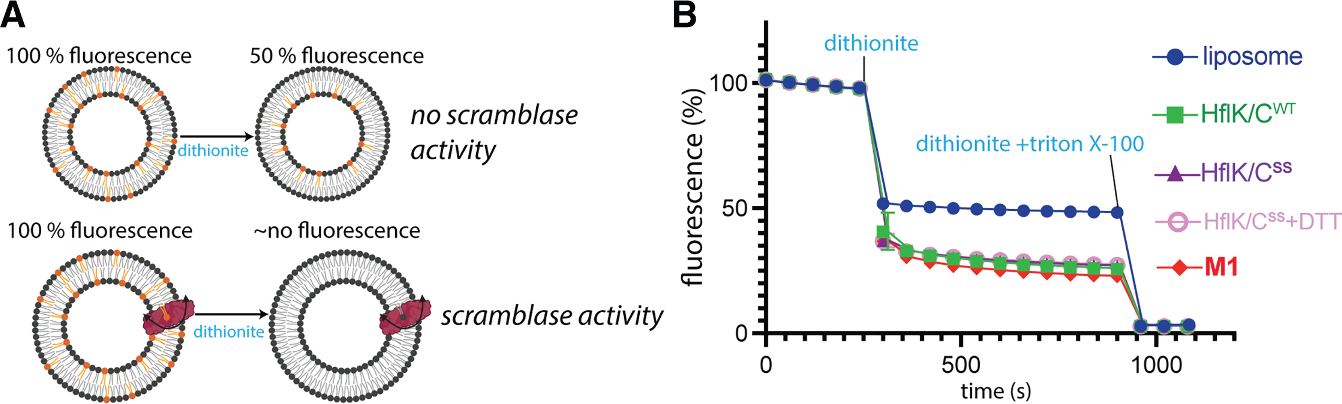
Scramblase activity assay of HflK/C^SS^ in the presence or absence of DTT, the M1 mutant, and wild-type HflK/C (A) Schematic representation of the scramblase activity assay (partially created with BioRender.com). In a protein-free liposome (top), addition of dithionite quenches only NBD fluorophores in the outer leaflet, producing ~50% fluorescence loss. In the presence of an active scramblase (bottom), NBD-labeled lipids are translocated between leaflets, continuously exposing inner-leaflet NBD-labeled lipids to the outer leaflet, where they are quenched by dithionite, resulting in >50% fluorescence loss. (B) HflK/C^SS^ in the closed (−DTT) and open (+DTT) conformations, as well as the M1 mutant, shows greater than 50% fluorescence reduction, indicating lipid scramblase activity similar to that of wild-type HflK/C, whereas liposomes alone show only ~50% quenching. All assays were performed using a 50 nM complex (as determined by a BSA assay through quantification of the HflK band) at 30°C. Values represent means (*n* = 3 technical replicates), with error bars indicating the SD.

Because both the crosslinked HflK/C^**SS**^ and the **M1** mutant failed to support bacterial growth under tobramycin stress, we sought to determine whether these mutations caused a global loss of HflK/C activity or instead impaired a specific function required under aminoglycoside stress. To address this, we assessed scramblase activity, a membrane-remodeling function of the FtsH·HflK/C complex that we previously characterized.^4^ The HflK/C complex enhances lipid flip-flop across membranes, which can be quantified using a fluorescence-based scramblase assay.^4,35,36^ In this assay, NBD-labeled lipids are symmetrically distributed between the inner and outer leaflets of liposomes. In the absence of an active scramblase, addition of dithionite, which cannot cross the lipid bilayer due to its negative charge, quenches NBD fluorophores in the outer leaflet only, resulting in an ~50% reduction in fluorescence. In the presence of an active scramblase, rapid exchange between the two leaflets exposes additional NBD-labeled lipids to dithionite, leading to a greater reduction in fluorescence.^36^ The HflK/C^**SS**^, both in the presence and absence of 5 mM DTT, as well as the **M1** mutant, showed scramblase activity similar to that of the WT complex (Figure 5). These results demonstrate that neither crosslinking nor disruption of FtsH binding compromises the membrane-remodeling function of HflK/C.

### The HflK/C^WT^ adopts a new conformation under antibiotic stress

Since the fully closed HflK/C assembly and mutations that disrupt FtsH-HflK/C interactions led to reduced bacterial growth under antibiotic stress, we conclude that the flexible, open conformation of HflK/C and its interaction with FtsH are essential for regulating FtsH proteolysis during tobramycin-induced stress. To investigate how the HflK/C complex structurally aids bacterial growth under antibiotic stress, we exposed bacteria to tobramycin and subsequently isolated the endogenous FtsH complex using either the micelle-forming detergent DDM or, in a detergent-free approach, the nanodisc-forming polymer carboxy-diisobutylene-maleic acid (Carboxy-DIBMA), which we have previously employed for FtsH·HflK/C structural studies.^4^ Under normal, non-stressed growth conditions, our previous structural analyses revealed an open, nautilus-like HflK/C assembly containing two FtsH hexamers within the cage, with one hexamer exhibiting lower occupancy, indicating that the complex can coexist with either one or two FtsH hexamers per assembly.^4^ Notably, previous studies of FtsH·HflK/C complexes obtained through protein overproduction also reported these open assemblies as a predominant state and a closed conformation as a minor population; however, the open form was interpreted as a rupture-like structure arising from purification artifacts.^21^

**Figure 6.**
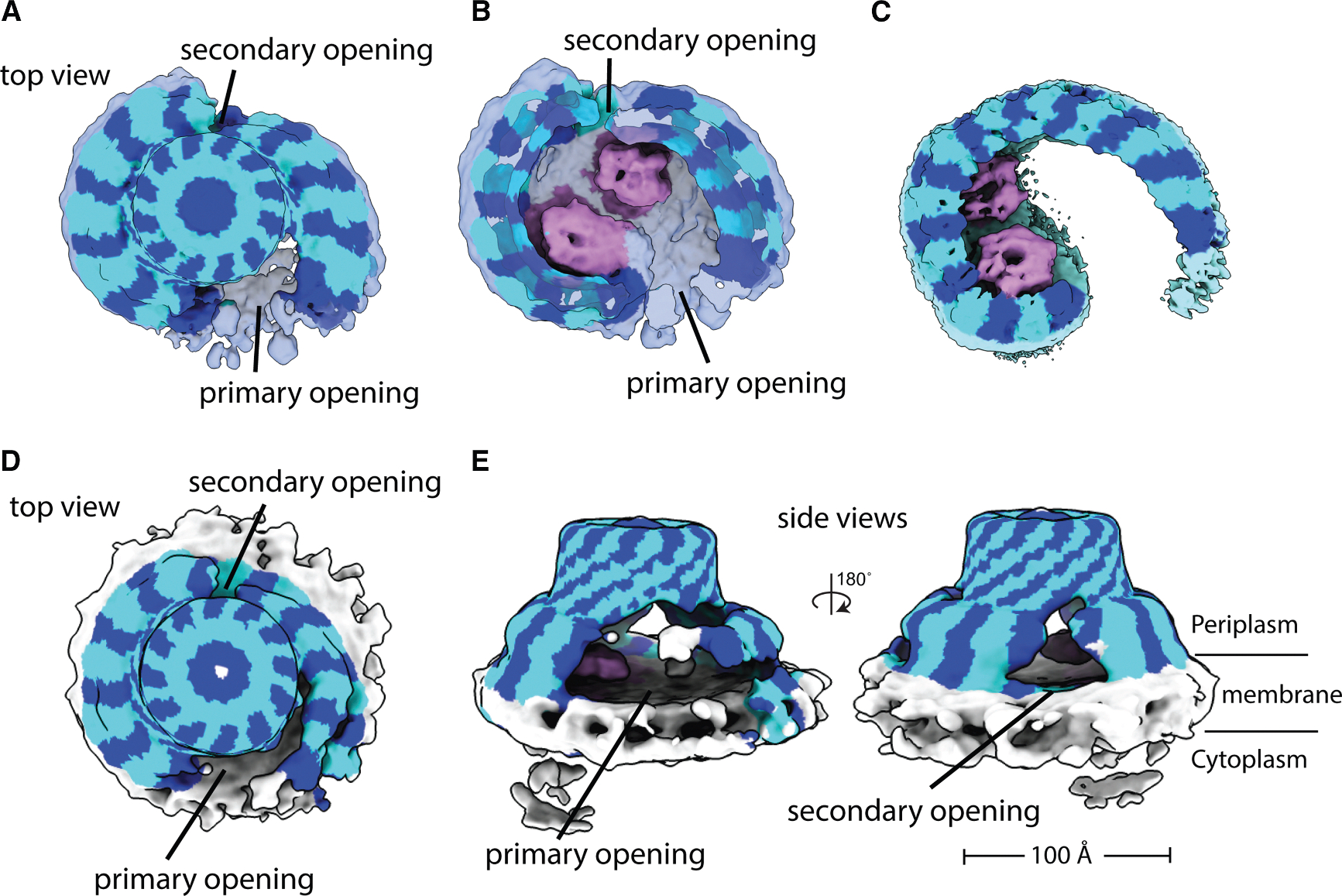
Cryo-EM maps of the native HflK/C complex from cells treated with tobramycin The maps are color coded using an atomic model: HflK in blue, HflC in cyan, and FtsH in pink. (A and B) Cryo-EM reconstructions of DDM-solubilized HflK/C complexes showing a top view and a sliced top view, respectively. Two openings are observed near the positions where FtsH hexamers are embedded within the HflK/C assembly. (C) Previously determined cryo-EM map of the nautilus-like FtsH·HflK/C complex (EMD-46057). (D and E) Cryo-EM maps of HflK/C complexes extracted into Carboxy-DIBMA nanodiscs, showing the secondary opening toward the lipid bilayer, as seen from both top (D) and side (E) views. Notably, this map reveals the lipid bilayer surrounding the complex and shows only one strong FtsH periplasmic domain density compared with the DDM-solubilized samples.

Surprisingly, structural analysis of complexes purified from tobramycin-treated cells (Figures S13–S16; Table 1) revealed a secondary opening in the HflK/C assembly, located opposite the previously identified primary aperture and positioned near the second FtsH hexamer (Figure 6). The width of this opening (~30–50 Å) is narrower than that of the initial aperture (~70–100 Å) yet still sufficiently wide to permit the diffusion of small membrane proteins into the assembly. This secondary opening was observed in both DDM micelles and Carboxy-DIBMA nanodiscs; however, only the latter additionally revealed the presence and orientation of the adjacent lipid bilayer. In addition to the density corresponding to the periplasmic side of FtsH, we also observed extra density surrounding FtsH, which we suspected represented substrate. However, the resolution of these maps (~9–11 Å) was insufficient to draw definitive conclusions. Notably, in a subset of particles from previously published overproduced FtsH·HflK/C datasets, a similar dual-opening HflK/C arrangement was observed; however, these classes were discarded during the final rounds of classification and were therefore not further analyzed.^21^ Importantly, this dual opening was not observed in previous cryoDRGN analysis of endogenous complexes extracted from cells under normal growth conditions,^4^ indicating that it is either extremely rare or not populated in unstressed conditions. Its appearance in the overproduction datasets of Ma et al. may therefore reflect, at least in part, enrichment of a minor native state and/or induction of membrane stress resulting from protein overproduction.

To further evaluate the structural heterogeneity of our complex from tobramycin-treated cells, we performed cryoDRGN analysis,^37^ which revealed variability in both the aperture widths and the positioning of FtsH within the assembly (Video S1). These results indicate that tobramycin-treated samples comprise a continuum of related conformational states rather than discrete, well-defined populations, which likely contributes to the limited resolution of the final reconstructions (Figures S13–S16).

## DISCUSSION

The HflK/C membrane assembly, a member of the SPFH protein family, and the AAA+ protease FtsH together play a key role in bacterial stress responses, particularly in mediating resistance to aminoglycosides.^17,18^ Despite their importance, the mechanisms by which HflK/C support bacterial recovery by modulating FtsH activity remain poorly understood.^18,21^ Additionally, recent structures of HflK/C in two distinct conformations—an asymmetric, open, nautilus-like structure^4^ and a symmetric, closed, cage-like structure^21,22^—have raised new questions about the biologically active conformation of HflK/C and its role in regulating the proteolytic activity of FtsH. By engineering disulfide bonds into the hat region of HflK/C, we were able to stabilize the closed conformation (Figures 1 and 2). Cryo-EM analysis revealed that the closed conformation observed in this crosslinked variant of HflK/C resembles the previously reported open structure, except for subunits located at the entrance of the opening (Figures 3A and 3B). Importantly, formation of the closed cage does not interfere with FtsH binding (Figure 3D), and we confirmed that the assembly contains at least one fully ordered FtsH molecule (Figures 3A, S4, and S5). The closed conformation reported here also shares similarities with closed assemblies of other SPFH family member structures^23–25^ but differs from the previously reported closed FtsH·HflK/C complex^22^ (Figures 3B–3D and S6). We propose that this discrepancy may stem from the use of a non-specific crosslinker and/or imposition of C_4_ symmetry in the previous study,^22^ which could have altered the arrangement of the SPFH domains and resulted in a cubic cage structure rather than the rounded one observed here (Figures 3 and S6). Our phenotypic results showed that HflK/C in the closed conformation significantly impairs bacterial growth under aminoglycoside stress (Figures 4A, 4B, and S12), suggesting that this conformation represents an inactive state of the HflK/C assembly during membrane stress. Importantly, both the crosslinked HflK/C and the **M1** mutant retain scramblase activity similar to that of the WT, indicating that the observed growth defects do not arise from a global loss of HflK/C activity (Figure 5). However, our phenotypic assay did not reveal any growth defect under normal conditions for the fully closed variant of HflK/C (Figure 4A), which is consistent with the fact that *hflK* and *hflC* are non-essential under standard growth conditions.^4,17^

While the question of how the HflK/C assembly contributes to substrate recruitment by FtsH remains open, our mutational analysis of HflK suggests that specific interactions between FtsH and HflK are critical for regulating FtsH proteolysis to support bacterial growth under aminoglycoside stress (Figures 4C–4E, S11, and S12). These interactions may facilitate the spatial organization of FtsH and its substrates within the HflK/C complex, potentially enhancing substrate recognition by the AAA domain through an increased local concentration of substrates around FtsH hexamers. Consistent with this model, the cryo-EM structure of the native FtsH·HflK/C complex isolated from tobramycin-treated cells revealed an unprecedented opening near the second FtsH (Figure 6). This aperture likely facilitates access to FtsH molecules under proteotoxic stress, allowing excess unfolded protein substrates to enter the microdomain from multiple directions. Whether this opening results from post-translational modifications or from proteolytic cleavage by endopeptidases or arises passively in response to oxidative membrane damage^18^ remains to be determined. This result, together with our previous proteomic analysis,^4^ supports a model in which dynamic opening of HflK/C fine-tunes FtsH-mediated proteolysis, particularly during aminoglycoside-induced membrane stress, when misfolded or damaged membrane proteins accumulate and must be rapidly cleared by the FtsH to restore cellular homeostasis.^18,38^

Interestingly, other members of the SPFH family have also been implicated in stress recovery mechanisms across diverse organisms.^20,39–42^ For instance, stomatin contributes to mitigating LPS-induced oxidative stress and inflammation in the mouse lung^42^; flotillins stabilize unfolded proteins in methicillin-resistant *Staphylococcus* under cellular stress^41^; and prohibitin serves as a biomarker of oxidative stress in ocular tissues.^39,43^ Given the shared structural and functional features, could the stress-induced structural changes observed in HflK/C represent a conserved property among SPFH family members across species?

Recent cryo-EM structures of other SPFH proteins have predominantly revealed closed assemblies.^21–26,44^ However, the predominance of closed conformations observed in other SPFH proteins may, in part, reflect experimental factors such as protein overexpression,^21,22,26^ the imposition of high symmetry during 3D cryo-EM reconstruction (often applied to a subset of symmetrical particles following extensive 3D classifications),^21,26^ or using non-specific crosslinkers,^22^ all of which could bias structural determination toward a closed state. Another possibility is that the conformational flexibility observed in HflK/C may represent a unique feature of SPFH complexes that interact with AAA+ proteases. Supporting this notion, a recent paper using *in situ* cryo-electron tomography (cryo-ET) of mitochondria under depolarizing conditions showed that prohibitins, the mitochondrial homologs of HflK/C, can adopt both an asymmetric, open, nautilus-like conformation and a symmetric, closed arrangement^45^ similar to our closed HflK/C structure presented here, although m-AAA was not observed in those assemblies. Our use of targeted disulfide crosslinking to selectively stabilize the closed HflK/C conformation and demonstrate its physiological impact provides an approach that could be extended to other SPFH family members to probe the biological relevance of the closed state. Overall, our study provides novel insights into how HflK/C conformational dynamics contribute to bacterial recovery from aminoglycoside-induced stress and reveals how this massive membrane complex structurally adapts to proteotoxic conditions. Our study may provide the ground-work for understanding the functional mechanisms of SPFH family proteins in both bacteria and eukaryotes, as well as their roles in cellular stress response pathways.

### Limitations of the study

Although stabilizing the closed HflK/C conformation impaired recovery from aminoglycoside-induced stress, we did not directly test whether enforcing a constitutively open HflK/C state would also affect cellular fitness. Addressing this question will require additional engineering strategies or small molecules capable of selectively stabilizing the open conformation. In addition, pronounced conformational heterogeneity of the FtsH AAA+ domain limited our ability to determine how the closed HflK/C assembly alters FtsH structure or proteolytic mechanisms at high resolution. Finally, the stress-induced dual-opening HflK/C conformation observed in complexes isolated from tobramycin-treated cells was resolved at low resolution and exhibited substantial heterogeneity, most likely reflecting mixed conformational states and diverse substrate populations. This inherent heterogeneity prevented confident assignment of putative substrate density, precluded determining whether the additional opening results from proteolytic cleavage, post-translational modification, or passive membrane damage associated with aminoglycoside stress, and complicated the design of biochemical assays to directly assess how the dual-opening state influences FtsH proteolytic activity.

## RESOURCE AVAILABILITY

### Lead contact

Requests for further information and resources should be directed to and will be fulfilled by the lead contact, Alireza Ghanbarpour (alirezag@wustl.edu).

### Materials availability

All plasmids and bacterial strains generated in this study are available from the lead contact upon reasonable request. Any restrictions on material transfer will be subject to institutional materials transfer agreement (MTA) requirements.

### Data and code availability

- Cryo-EM density maps and corresponding atomic coordinates generated in this study have been deposited in the PDB under accession number PDB: 9PAW and in the Electron Microscopy Data Bank (EMDB) under accession numbers EMD-71448, EMD-71449, and EMD-71447. RNA-seq data generated in this study have been deposited in the Sequence Read Archive (SRA) under BioProject accession number PRJNA1282740. All other data supporting the findings of this study are available within the article and its supplemental information.
- This paper does not report original code.
- Any additional information required to reanalyze the data reported in this paper is available from the lead contact upon reasonable request.

## STAR★METHODS

### EXPERIMENTAL MODEL AND STUDY PARTICIPANT DETAILS

#### Bacterial strains

This study used bacterial experimental models only and did not involve human participants, animals, plants, primary tissues, or cell lines. The bacterial strains used were *Escherichia coli* BL21(DE3) Δ*hflKC* carrying a chromosomally encoded C-terminal FLAG tag on FtsH, and *E. coli* MG1655 Δ*hflKC*. The BL21(DE3) Δ*hflKC* FtsH-FLAG strain was described previously,^4^ and the MG1655 Δ*hflKC* strain was a generous gift from the Sourjik laboratory.^19^

#### Experimental model characteristics relevant to this study

The experimental models in this work were bacterial strains. Sex, gender, ancestry, race, and ethnicity are therefore not applicable to this study. No mammalian or other eukaryotic cell lines were used in this study; therefore, cell line authentication and mycoplasma testing are not applicable.

#### Biosafety and oversight

All experiments were performed using standard laboratory strains of *E. coli* and recombinant DNA methods in accordance with institutional biosafety practices and approved laboratory procedures at Washington University School of Medicine.

### METHOD DETAILS

#### Plasmid construction

Plasmids were generated by PCR amplification of the region spanning 170bp upstream of *hflK* to 51bp downstream of *hflC* from the *E. coli* BL21 chromosome and cloning into a plasmid containing a propionate-inducible promoter.^47^ Additional mutations corresponding to the HflK/C^**SS**^ variant (A270C and A283C in HflK; A233C and A264C in HflC) and the **M1** variant of HflK (R141A, E142A, R185A) were introduced using synthetic gene blocks (GenScript Inc.) and assembled by Gibson assembly. A C-terminally His-tagged version of *hflC* was generated by Q5 site-directed mutagenesis following the manufacturer’s protocol.

#### FtsH⋅HflK/C purification

Previously constructed strains containing a C-terminal FLAG-tagged variant of FtsH,^4^ introduced at the endogenous *ftsH* locus in *E. coli* BL21(DE3) or BL21(DE3) Δ*hflK/C*, were used for all pull-down experiments. A single bacterial colony was inoculated into 50 mL of LB medium containing 50 μg/mL kanamycin and 35 μg/mL chloramphenicol and grown overnight at 37°C with shaking. Overnight cultures were inoculated into 4 L of fresh medium (50% LB:50% YT) and incubated at 37°C with shaking at 220 rpm for 24 h. Cells were harvested by centrifugation at 4,000 rpm for 25 min at 4°C using an H-1200 rotor. The resulting pellet was resuspended in Buffer A (100 mM KCl, 50 mM Tris-HCl, pH 8.0, 10% glycerol, 5 mM MgCl_2_, 100 μM ZnCl_2_) and stored at −80°C until further use. For lysis, frozen cells were thawed on ice and disrupted by sonication (30% amplitude, 10 s on/30 s off cycles, for a total of 3 min). Cell debris was removed by centrifugation at 14,000 rpm for 20 min at 4°C using a JL20 rotor. The supernatant was ultracentrifuged at 30,000 rpm for 1 h at 4°C in a 45 Ti rotor to isolate the membrane fraction. The resulting membrane pellet was resuspended in Buffer A and homogenized using a Dounce homogenizer. A 2% DDM stock solution was added to achieve a final DDM concentration of 1%, and the mixture was incubated at 4°C for 2 h to allow membrane protein solubilization. Samples were then ultracentrifuged at 30,000 rpm for 1 h at 4°C, and the clarified supernatants were collected and kept on ice. The samples were incubated with pre-washed M2-FLAG resin (Millipore Sigma, Cat. #A2220). The solubilized protein solution was added to the prepared resin and incubated at 4°C for 2 h with gentle rotation. After incubation, the resin was centrifuged at 400 *g* for 5 min, loaded into a gravity column, and washed with 2 mL of Buffer A (in 200 μL aliquots) containing 0.03% DDM. A second wash was performed using Buffer B (400 mM NaCl, 50 mM Tris-HCl, 5 mM MgCl_2_, 10% glycerol, and 0.03% DDM). The samples were eluted using a 0.25 mg/mL Flag peptide (APEXBio) in Buffer B (100 mM KCl, 50 mM Tris-HCl pH 8.0, 5% glycerol, 5 mM MgCl_2_, 100 μM ZnCl_2_, 0.03% DDM). The pooled elution fractions were concentrated using a 100 kDa cutoff Centricon filter (Millipore Inc.). The same protocol was used for GDN sample preparation, except 2% GDN was used for membrane solubilization and 0.03% GDN was employed for the wash and elution steps. For carboxy-DIBMA and DDM extractions from tobramycin-treated cells, the previously described protocol was followecd.^4^ The only difference was that cells were treated with 2.5 μg/mL tobramycin overnight before complex extraction and purification.

#### Phenotypic assay

Cellular assays were performed using previously constructed *E. coli* BL21(DE3) Δ*hflKC* cells carrying a chromosomally encoded C-terminal FLAG tag at the *ftsH* locus^4^ or MG1655 Δ*hflKC* cells.^19^ All plasmids, or an empty vector control, were transformed into BL21(DE3) Δ*hflKC* or MG1655 Δ*hflKC* cells and assayed as described below. For growth assays, overnight cultures were grown at 37°C and then diluted to an A_600_ of ~0.1 in LB medium containing chloramphenicol (35 μg/mL) and 1 mM sodium propionate, with or without tobramycin. Aliquots (200 μL) of bacterial suspensions containing different *hflK/C* variants or an empty vector were transferred to a 96-well clear flat-bottom microplate (Corning Inc.). Absorbance at 600 nm (A_600_) was measured every 5 min using a SpectraMax M2 plate reader.

#### Pull-down assay

Overnight cultures of cells expressing either wild-type or mutant constructs (using the same plasmids employed for structural and phenotypic assays) were inoculated into 1 L of fresh medium consisting of a 1:1 mixture of LB and YT. Cultures were incubated at 37°C with shaking for 24 h. Cells were harvested by centrifugation at 4,000 rpm for 30 min at 4°C, and cell pellets were resuspended in 30 mL of buffer A and stored at −80°C. Upon thawing, cells were lysed by sonication (30% amplitude, 10 s on/30 s off, total sonication time of 3 min). The lysate was centrifuged at 14,000 *g* for 30 min using a JA-20 rotor to remove cell debris. The cleared supernatant was then ultracentrifuged at 30,000 rpm for 1 h in a Ti-45 rotor to isolate the membrane fraction. The membrane pellet was resuspended in 6 mL of Buffer A and homogenized with 5–6 strokes of a Dounce homogenizer. An equal volume of 2% DDM was added to reach a final concentration of 1% DDM, and the suspension was incubated at 4°C for 2 h with gentle mixing to solubilize membrane proteins. Following solubilization, the sample was ultracentrifuged again at 30,000 rpm using 45Ti rotor for 1 h, and the supernatant containing solubilized proteins was collected and kept on ice. For affinity purification, 250 μL of M2-FLAG resin was washed with 2 mL Buffer A containing 0.03% DDM and pelleted by centrifugation at 400*g* for 5 min. The solubilized protein solution was incubated with the prewashed resin (with buffer A) at 4°C for 2 h with gentle mixing. After incubation, the resin was pelleted by centrifugation at 400*g*, transferred to a gravity-flow column, and washed sequentially with 2 mL Buffer A (+0.03% DDM) and 2 mL Buffer B (+0.03% DDM). Elution was performed using 0.25 mg/mL Flag peptide (APEXBio) diluted in Buffer C (100 mM KCl, 50 mM Tris-HCl pH 8.0, 5% glycerol, 5 mM MgCl_2_, 100 μM ZnCl_2_, 3 mM β-mercaptoethanol, 0.03% DDM). Eluates were pooled and concentrated using a 100 kDa cutoff centrifugal filter unit (centrifuged at 6,000 rpm at 4°C). For SDS-PAGE analysis, 4 μL of each sample fraction was mixed with 10 μL of 4× Laemmli buffer, boiled for 5 min, and 6 μL of the mixture was loaded onto the 4–20% Mini-PROTEAN TGX Precast Protein Gels.

#### Cryo-EM sample preparation

For the cross-linked FtsH·HflK/C (FtsH·HflK/C^**SS**^) structure, samples were prepared by applying 2.5 μL of ~0.3 mg/mL of FtsH·HflK/C (crosslinked) complex on 200 mesh Quantifoil 2 nm carbon 2/1 copper grids. The grids were glow-discharged using a GloQube Plus (MiTeGen) at 15 mA for 20 s. Sample loaded grids were blotted for 4s with a blot force of +4 at 6°C and 100% relative humidity using a FEI Vitrobot Mark IV instrument (Thermo Scientific).

For the DDM-solubilized FtsH·HflK/C complex extracted from the tobramycin treated cells, ~0.7 mg/mL HflK/C sample was used for grid preparation. 2-nm carbon-supported 200-mesh Quantifoil 2/1 copper grids, which had been glow-discharged for 20 s in an easiGlow glow discharger (Pelco) at 15 mA, were utilized. For the Carboxy-DIBMA-extracted sample from the tobramycin treated cells, a concentration of ~0.3 mg/mL of the FtsH·HflK/C complex was applied to 2-nm carbon-supported 200-mesh Quantifoil 2/1 copper grids, also glow-discharged for 20 s in an easiGlow glow discharger (Pelco) at 15 mA, and the sample was vitrified as above.

#### Cryo-EM data collection

For the cross-linked FtsH·HflK/C (FtsH·HflK/C^**SS**^) complex structure, 8,766 movies were collected with EPU on a Titan Krios G3 using multiple images per hole with an acceleration voltage of 300 kV and magnification of 75 k, detected on a Falcon 4 detector for an effective pixel size of 0.8654 Å. Movies were collected as 50 frames with a defocus range from −1.2 to −2.4 μm and a total exposure per specimen of 52.68 e^−^/Å^2^.

For the DDM-solubilized FtsH·HflK/C complex extracted from the tobramycin treated cells, 21,895 movies were collected with EPU using aberration-free image shift (AFIS) and hole-clustering method on a Titan Krios G3i with an acceleration voltage of 300 kV and magnification of 130,000×, detected in super-resolution mode on a Gatan K3 detector for an effective pixel size of 0.654 Å (binned by 2). Movies were collected as 40 frames with a defocus range from −0.5 to −1.75 μm and a total exposure per specimen of 48.02 e^−^/Å^2^.

For the Carboxy-DIBMA-extracted FtsH·HflK/C sample from tobramycin-treated cells, 32,694 movies were collected as 40-frame, with a defocus range of −1.3 μm and a total electron dose of 46.86 e^−^/Å^2^ per specimen. Data acquisition was performed using AFIS hole clustering at a 25° stage tilt on a Titan Krios G3i operated at 300 kV acceleration voltage and 130,000× magnification. Images were recorded in super-resolution mode on a Gatan K3 detector, yielding an effective pixel size of 0.654 Å (binned by 2).

#### Cryo-EM pre-processing and particle picking

For the DDM-solubilized FtsH·HflK/C^**SS**^ complex, data processing was performed using cryoSPARC (v4.6) and default parameters unless noted. Raw movies (8,766) were pre-processed using “Patch motion correction”, and “Patch CTF estimation”. Particles (429, 520) were picked using the “Blob-picker” tool applied to 3000 micrographs selected at random. Particles were extracted (box size 640 px, Fourier cropped to 360 px) and classified using the ‘2D classification’ utility. The entire set of micrographs were then picked with the “Template picker” tool, using classes from 2D classification. 1,866,826 particles were extracted (box size 600 px, Fourier cropped to 256 px) and subjected to multiple rounds of 2D classification, resulting in the selection of 309,350 particles as a preliminary stack.

For the DDM-solubilized FtsH·HflK/C complex extracted from the tobramycin treated cells, data processing was performed using cryoSPARC (v4.5^49^ and default parameters unless noted. Raw movies (21,895) were pre-processed using “Patch motion correction”, and “Patch CTF estimation”. Particles (1,089,115) were picked using the “Blob-picker” tool applied to 3000 micrographs selected at random. After two rounds of 2D classification, 38 2D classes were selected as a template. Particles were picked using “Template picker” and were extracted (box size 900 px, Fourier cropped to 220 px) and classified using the ‘Ab-initio’ utility (3 classes). 382,188 particles were selected and subjected to multiple rounds of 2D classification, resulting in the selection of 146,192 particles as a preliminary stack. For the Carboxy-DIBMA-extracted FtsH·HflK/C sample from tobramycin-treated cells, 32,694 movies were pre-processed using “Patch motion correction” and “Patch CTF estimation.” A total of 2,462,066 particles were initially picked using the “Blob picker.” After multiple rounds of 2D classification, three 2D classes were selected as templates. Particles were then re-picked using the “Template picker” and extracted (box size: 900 px, Fourier cropped to 128 px). After four additional rounds of 2D classification, 43,479 particles were selected as the preliminary stack.

#### Ab initio reconstruction and global refinement

For the FtsH·HflK/C^**SS**^ complex solubilized in DDM, “Ab-initio reconstruction” using 1 class was performed, resulting in a model and the particle stack (300,000 particles) was selected for subsequent “non uniform refinement”. Particles were then re-extracted using 512 px box size down sampled to 384 px. Extracted particles were then run through non uniform refinement with C_4_ symmetry applied followed by another round of nonuniform refinement with C_1_ symmetry. The particles were then run through local refinement with C_1_ symmetry. A mask was then created in ChimeraX and local refinement was performed using this mask. After applying global and local CTF refinement followed by local refinement with C_1_ symmetry, the final map achieved at GSFSC resolution of ~2.93 Å after FSC mask auto-tightening. Model building was carried out using Chimera^50^ (v1.9), Coot^51^ (v0.9.4), and Phenix^52^ (v1.21). Because FtsH was not well resolved in the final reconstructions, we undertook additional focused classification and refinement strategies (Figure S4). We first generated a cylindrical mask encompassing the expected position of FtsH and performed 3D classification into 15 classes without pose refinement. Volumes exhibiting density features consistent with FtsH were selected and used as initial models (six classes) for heterogeneous refinement. Classes showing discernible FtsH density were subsequently subjected to an additional round of 3D classification. From this step, a single volume was selected and duplicated six times to seed another round of heterogeneous refinement (using all particles), which resulted in improved visualization of the periplasmic domain of FtsH. Particles corresponding to assemblies containing FtsH and adopting a fully closed HflK/C conformation were then selected for further analysis. Despite these efforts, local refinement of the full complex did not yield strong FtsH density, suggesting that alignment was dominated by the rigid HflK/C scaffold. To overcome this bias, we generated two separate masks: one encompassing only the HflK/C assembly and another containing only the periplasmic domain of FtsH. Using the HflK/C-only mask, we performed signal subtraction followed by homogeneous refinement. The resulting particle stack was then subjected to local refinement using the FtsH periplasmic mask, yielding a reconstruction at approximately 4 Å resolution. The resulting density clearly resolved the periplasmic domain of FtsH, and structural superposition with the previously determined model (PDB ID: 9CZ2) revealed no detectable conformational differences (Figure S5).

For the DDM-solubilized FtsH·HflK/C complex extracted from tobramycin-treated cells, *ab initio* reconstruction with three classes was performed. One particle class (58,175 particles) was selected for subsequent non-uniform refinement followed by local refinement. The final map achieved at GSFSC resolution of 9.1 Å after FSC mask auto-tightening. For the Carboxy-DIBMA-extracted FtsH·HflK/C sample from tobramycin-treated cells, *ab initio* reconstruction was performed using two classes. The class showing the FtsH·HflK/C complex was selected for non-uniform refinement leading to the final map achieved a GSFSC resolution of ~11.5 Å after FSC mask auto-tightening.

#### Scramblase activity assay

The scramblase activity assay was performed using a previously described method.^4,35^ Briefly, lipids (POPC, POPE, and NBD-PE) were purchased from Avanti Polar Lipids and used to prepare liposomes. A lipid mixture consisting of POPC (90% w/w), POPE (9.5%), and NBD-PE (0.5%), all dissolved in chloroform, was combined and dried under a gentle stream of argon. The dry lipid film was hydrated in buffer (50 mM HEPES, pH 7.6, 200 mM NaCl) to a final lipid concentration of 10.5 mM. The suspension was incubated at 37°C for 10 min, followed by resuspension and 10 freeze–thaw cycles. The lipid mixture was then extruded 30 times through a 400 nm polycarbonate filter. Liposomes (total volume 250 μL, lipid concentration 5.25 mM) were destabilized by the addition of Triton X-100 and incubated at room temperature for 1.5 h. Proteins were then added, and the mixture was rotated at room temperature for an additional 1 h. Pre-washed Bio-Beads (20 mg) were added, and the mixture was rotated at room temperature for 1 h, followed by the addition of a second aliquot of Bio-Beads (20 mg) and incubation for 2 h at room temperature. The mixture was subsequently transferred to a new tube containing fresh Bio-Beads (40 mg) and rotated overnight at 4°C. Bio-Beads were then carefully removed. Lipid scramblase activity was measured at 30°C in 96-well plates containing 100 μL of either liposomes or proteoliposomes. Fluorescence was monitored using a BioTek Synergy HTX multi-mode plate reader by following the time-dependent decrease in NBD fluorescence (excitation 460 nm; emission 538 nm) after addition of sodium dithionite to a final concentration of 5 mM. Triton X-100 (0.5%) was finally added as a control to fully solubilize the liposomes and ensure complete NBD reduction.

#### Western blot analysis

Western blot was performed by pelleting 1 mL of cells expressing either the HflK/C^**WT**^ or **M1** mutant at an *A*_600_ of 3.0 and 3.2 via centrifugation at 17000*g* for 3 min. The supernatant was discarded, and cell pellets were resuspended in Laemmli buffer, supplemented with 1× EDTA-free protease inhibitor cocktail (Complete, Roche). Samples were boiled for 5 min and analyzed by SDS-PAGE. The bands were transferred onto a pre-activated PVDF membrane using the Trans-Blot Turbo transfer system (Bio-Rad). The membrane was incubated with 6x-His Tag Monoclonal Antibody (Thermo Fisher Scientific Inc.;1:5000 dilution), followed by goat anti-mouse IgG secondary antibody (Thermo Fisher Scientific Inc; 1:10,000 dilution). Signal was detected using Clarity Western ECL substrate (Bio-Rad) and visualized with a Bio-Rad imaging system.

#### RNA-seq sample preparation and analysis

*E. coli* BL21 cells expressing FLAG-tagged wild-type FtsH were grown to an A_600_ of 0.5 in the presence or absence of 2.5 μg/mL tobramycin. Total RNA was extracted using the RNeasy Plus Mini Kit (Qiagen, cat. no. 74134) and processed at the Genome Technology Access Center, McDonnell Genome Institute, Washington University School of Medicine. RNA-seq libraries were prepared following the manufacturer’s protocol, indexed, pooled, and sequenced on an Illumina NovaSeq X Plus. Base calling and demultiplexing were performed using Illumina DRAGEN and BCLconvert (v4.2.4).

Reads were aligned to the primary genome assembly using STAR (v2.7.11 b).^53^ Gene-level counts were obtained using Subread:featureCounts (v2.0.8),^54^ and transcript-level quantification of known Ensemble isoforms was performed with Salmon (v1.10.0.^55^ Sequencing quality metrics, including total aligned reads, uniquely mapped reads, and detected features, were assessed. Ribosomal RNA content, splice junction saturation, and read distribution across gene models were evaluated using RSeQC (v5.0.4).^56^ Gene counts were imported into the EdgeR package (Bioconductor v4.4.0)^57^ and normalized using the trimmed mean of M-values (TMM) method. Genes corresponding to ribosomal RNAs and those not expressed at ≥1 count-per-million in at least three samples were excluded. The resulting TMM-normalized counts were analyzed using Limma.^58^ Weighted likelihoods, accounting for the mean-variance relationship across genes, were calculated with the voomWithQuality Weights function^59^ and used to fit a linear model. Model performance was assessed by plotting the residual standard deviation versus average log-counts, with a robustly fitted trend line. Differential expression was evaluated using empirical Bayes moderation, and significance was determined by applying Benjamini-Hochberg correction (FDR ≤0.05).

#### CryoDRGN analysis

To investigate structural heterogeneity, we analyzed all 41,259 particles from the Carboxy-DIBMA dataset using CryoDRGN v2.3.0.^37,60^ Particles were downsampled to a box size of 128 pixels (~5.1 Å/pixel) and used to train an eight-dimensional latent-variable model with 1024 × 3 encoder and decoder architectures. Particle poses and CTF parameters for CryoDRGN training were obtained from the non-uniform refinement. After 50 epochs of training, 100 volumes were sampled from the k-means cluster centers of the latent embeddings. Visual inspection of the resulting volumes, guided by our atomic model, revealed structural variability in the opening length, membrane span, and orientation of FtsH within the assembly and relative to the lipid bilayer.

### QUANTIFICATION AND STATISTICAL ANALYSIS

All statistical details for each experiment are provided in the corresponding figure legends. For experiments shown in the supplemental information, n and summary statistics are reported in the supplemental figure legends. For plate-reader-based growth assays, n represents independent biological replicates initiated from separate colonies. Unless otherwise stated, data are presented as mean ± SD. For RNA-seq analysis, differential expression was assessed from TMM-normalized counts using limma-voom, with significance determined after Benjamini–Hochberg multiple-testing correction at FDR ≤0.05. For volcano-plot visualization, genes were designated as significantly upregulated using the criteria described in the relevant figure legend and supplemental figure description.

## Supplementary Material

1

2

3

SUPPLEMENTAL INFORMATION

Supplemental information can be found online at https://doi.org/10.1016/j.celrep.2026.117231.

## Figures and Tables

**Table 1.** Cryo-EM data collection, processing, model building, and validation statistics

Name	Crosslinked HflK/C variant (HflK/C^SS^)	HflK/C^WT^ solubilized in DDM from tobramycin-treated cells	HflK/C^WT^ extracted in Carboxy-DIBMA from tobramycin-treated cells

PDB ID	9PAW	N/A	N/A
EMDB ID	EMD-71448	EMD-71449	EMD-71447
Microscope	Titan Krios G3	Titan Krios G3i	Titan Krios G3i
Camera	Falcon 4	K3 detector (Gatan)	K3 detector (Gatan)
Magnification	75,000×	130,000×	130,000×
Voltage (kV)	300	300	300
Total electron dose (e^−^/Å^2^)	52.68 e^−^/Å^2^	48.02 e^−^/Å^2^	46.86 e^−^/Å^2^
Defocus range (μm)	−1.2 to −2.4 μm	−0.5 to −1.75 μm	−1.3 μm
Pixel size (Å)	0.8654	0.654	0.654
Micrographs collected	8,766	21,895	32,694
Final particles	299,919	58,175	24,196
Symmetry	C_1_	C_1_	C_1_
Resolution (Å) FSC 0.143	2.9	9.1	11.5
Unmasked resolution (Å)	4.2	11	16
Sphericity score (out of 1)	0.85	0.90	0.80
All atoms	90,278	N/A	N/A
Protein residues	6,433	N/A	N/A
Ligands	12	N/A	N/A
Map-model CC (mask)	0.76	N/A	N/A
RMSD bond lengths (Å)	0.001	N/A	N/A
RMSD bond angles (deg.)	0.369	N/A	N/A
MolProbity score	1.18	N/A	N/A
Clash score	1.27	N/A	N/A
C-beta outliers (%)	N/A	N/A	N/A
Rotamer outliers (%)	0.00	N/A	N/A
Ramachandran favored (%)	95.18	N/A	N/A
Q-score (global/expected)	0.41/0.53	N/A	N/A

**KEY RESOURCES TABLE:** 

REAGENT or RESOURCE	SOURCE	IDENTIFIER
Antibodies		
6× Histag Monoclonal Antibody	Invitrogen	# MA1-21355; PRID: AB_557403
Goat anti-mouse IgG secondary antibody	Invitrogen	Cat# SA5-10276; RRID: AB_2868324
Bacterial and virus strains		
*E.Coli* BL21 ΔHflK/C FLAG tag on C terminus of FtsH on chromosome	Ghanbarpour et al.^4^	G2
MG1655 ΔHflK/C cells (a generous gift from Sourjik’s lab)	María Isabel Pérez-López et al., PLoS Biol; 23: e3003077.	G88
Chemicals, peptides, and recombinant proteins		
PrimeSTAR Max Ver.2	TaKaRa	#AO80337A
NEBuilder HiFi DNA Assembly Master Mix 10× KLD enzyme mix	NEW ENGLAND BioLabs NEW ENGLAND BioLabs	#M5520AA; #M0554
Chloramphenicol	GOLDBIO	# C-105-25
Kanamycin	GOLDBIO	# K-120-5
Tobramycin	Sigma-Aldrich	# T4014
Anti-FLAG M2 Affinity Gel	Sigma-Aldrich	# A2220
DYKDDDDK tag peptide	APExBIO	# A6002
LB Broth (Miller)	Sigma-Aldrich	# L3522
2xYT Medium	Sigma-Aldrich	# Y2627
Agarose	BIO-RAD	# 1613101
Clarity Max Western ECL Substrate	BIO-RAD	# 1705062
Potassium Chloride	Sigma-Aldrich	# P3911
Zinc Chloride	Sigma-Aldrich	# 208086
Magnesium Chloride	Sigma-Aldrich	# M1028
EveryBlot Blocking Buffer	BIO-RAD	#12010020
n-Dodecyl β-D-maltopyranoside (DDM)	GOLDBIO	# DDM5
GDN	Anatrace	# GDN101
Sodium Propionate	Sigma-Aldrich	# P1880
1-Palmitoyl-2-oleoyl-*sn*-glycero-3-phosphocholine	Avanti Research	# DO-008319
1,2-Dimyristoyl-*sn*-glycero-3-phosphoethanolamine-N-(7-nitro-2-1,3-benzoxadiazol-4-yl) (NBD-PE)	Avanti Research	# 810143
Surfact-Amps X-100	Thermo Fisher Scientific	# 28314
Critical commercial assays		
Gel extraction kit	QIAGEN	# 28704
RNeasy Plus Mini Kit	QIAGEN	# 74134
Plasmid isolation kit	QIAGEN	# 27106
Deposited data		
Electron Microscopy data	N/A	EMD-71448
Electron Microscopy data	N/A	EMD-71449
Electron Microscopy data	N/A	EMD-71447
Oligonucleotides and gene blocks		
HflK/C-F primer	GenScript Inc.	N/A
GAGCTCAGGAGGAATTGACG TAAGTCTGCAAGTTCG TATG CCGATCGTTGACT	N/A	N/A
HflK/C-R primer	N/A	N/A
CTAGAGGATCCCCGGGTACC TCATTATCCTTATAGA AAAAG AAAACCACCGAC	N/A	N/A
Gene block HflC	N/A	N/A
AAG GGC AAA GTT CCG GTC ATC AAC CCG AAC AGT ATG GCG GCG CTG GGT ATT GAA GTT GTC GAT GTG CGTATC AAG CAG ATC AAC CTG CCG ACC GAA GTG TCT GAA GCG ATC TAC AAC CGT ATG CGC GCC GAG CGT GAA TGCGTA GCG CGT CGT CAC CGT TCA CAA GGT CAG GAA GAA GCG GAA AAA CTG CGC GCG ACT GCC GAC TAT GAA GTGACC AGA ACG CTG GCA GAA TGC GAG CGT CAG GGC CGC ATC ATG CGT GGT GAA GGC GAT GCC GAA GCA GCC AAACTG TTT GCT GAT GCA TTC AGT AAA GAT CCG GAC TTC TAC GCA TTC ATC CGT AGC CTG CGT GCT TAT GAG AACAGC TTC TCT GG	GenScript Inc.	N/A
Gene block HflK	N/A	N/A
ATT CTG ACG GAA GGT CGT ACC GTG ATT CGT AGC GAT ACT CAG CGC GAA CTG GAA GAG ACG ATT CGT CCGTAT GAC ATG GGT ATC ACG CTG CTG GAC GTC AAC TTC CAG GCT GCT CGT CCG CCG GAA GAA GTA AAA GCG GCGTTT GAC GAT GCG ATT GCC GCG CGT GAA AAC GAA CAG CAA TAC ATT CGT GAA GCA GAA TGC TAT ACC AAC GAAGTT CAG CCG CGT GCG AAC GGT CAG TGC CAA CGT ATC CTC GAA GAG GCG CGT GCG TAC AAG GCC CAG ACC ATCCTG GAA GCT CAG GGT GAA GTG GCG CGC TTT GCT AAA CTT CTG CCG GAA TAT AAA GCC GCG CCG GAA ATT ACTCGC GAG CGT CTG TAT ATC GAG ACG ATG GAA AAA	GenScript Inc.	N/A
Gene Block HflK mutant	N/A	N/A
ACGAAGTCAAACCGGTGAACGTGGAA GCCGTGGCCGCCCTGGCCGCTTCTG GTGTGATGCTGACGTCGGACGAGAAC GTAGTGCGCGTTGAGATGAACGTGCA GTACCGCGTCACCAATCCGGAAAAAT ATCTGTATAGCGTGACCAGCCCGGAT GACAGCCTGGCCCAGGCTACCGACA GCGCCCTGCGTGGAGTT	N/A	N/A
HflC -Histag on c terminus, primer F	N/A	N/A
CACCACCACTAATATAACGACTGC GGTACAGGTC	GenScript Inc.	N/A
HflC -Histag on c terminus, primer R	N/A	N/A
ATGATGATGACGCGTTGCGGAAGTCGG	N/A	N/A
Recombinant DNA		
pPRO plasmid (Low copy number plasmid) was used for cloning WT HflK/C and generating all other mutants	Lee and Keasling^47^	Addgene Plasmid #17805
Software and algorithms		
ChimeraX-1.8	Goddard et al.^48^	https://www.rbvi.ucsf.edu/chimerax/
cryoSPARC (v4.7.0)	Punjani et al.^49^	https://cryosparc.com/
GraphPad Prism 10	N/A	https://www.graphpad.com/
EPU Data Collection Software	Thermo Fisher Scientific	https://www.thermofisher.com/ca/en/home/electron-microscopy/products/software-em-3d-vis/epu-software.html
Other		
PVDF membrane 0.2 μM	BIO-RAD	#1704156
Nano Drop one	Thermo Fisher Scientific	N/A
C1000 Touch Thermal Cycler	BIO-RAD	N/A
Trans-Blot Turbo Transfer System	BIO-RAD	N/A
ChemiDoc Imaging System	BIO-RAD	N/A
Amicon Ultra-0.5 Centrifugal Filters	Merck Millipore Ltd	UFC510096
R2/1 200 mesh grids	Quantifoil	Q2100CR1
Titan Krios 300 kV	Thermo Fisher Scientific	N/A
Talos Arctica 200 kV	Thermo Fisher Scientific	N/A
Vitrobot Mark IV system	Thermo Fisher Scientific	N/A

## Highlights

- Disulfide crosslinking enables control of HflK/C conformational states
- Stabilizing closed HflK/C compromises aminoglycoside recovery
- Stress recovery requires productive interactions between HflK/C and FtsH
- Aminoglycoside stress induces a novel HflK/C conformation with dual openings
