# Supplementary material for: Structural plasticity of the membrane-bound protein degradation assembly supports bacterial adaptation to stress
Source: Cell Rep. Author manuscript; Available in PMC 2026 May 29. (PMC13220708; doi:10.1016/j.celrep.2026.117231)
Supplement: 1 [file NIHMS2170795-supplement-1.pdf]

Cell Reports, Volume *45*

Supplemental information

Structural plasticity of the membrane-bound protein degradation assembly supports bacterial adaptation to stress

Naseer Iqbal, Sandro Keller, and Alireza Ghanbarpour

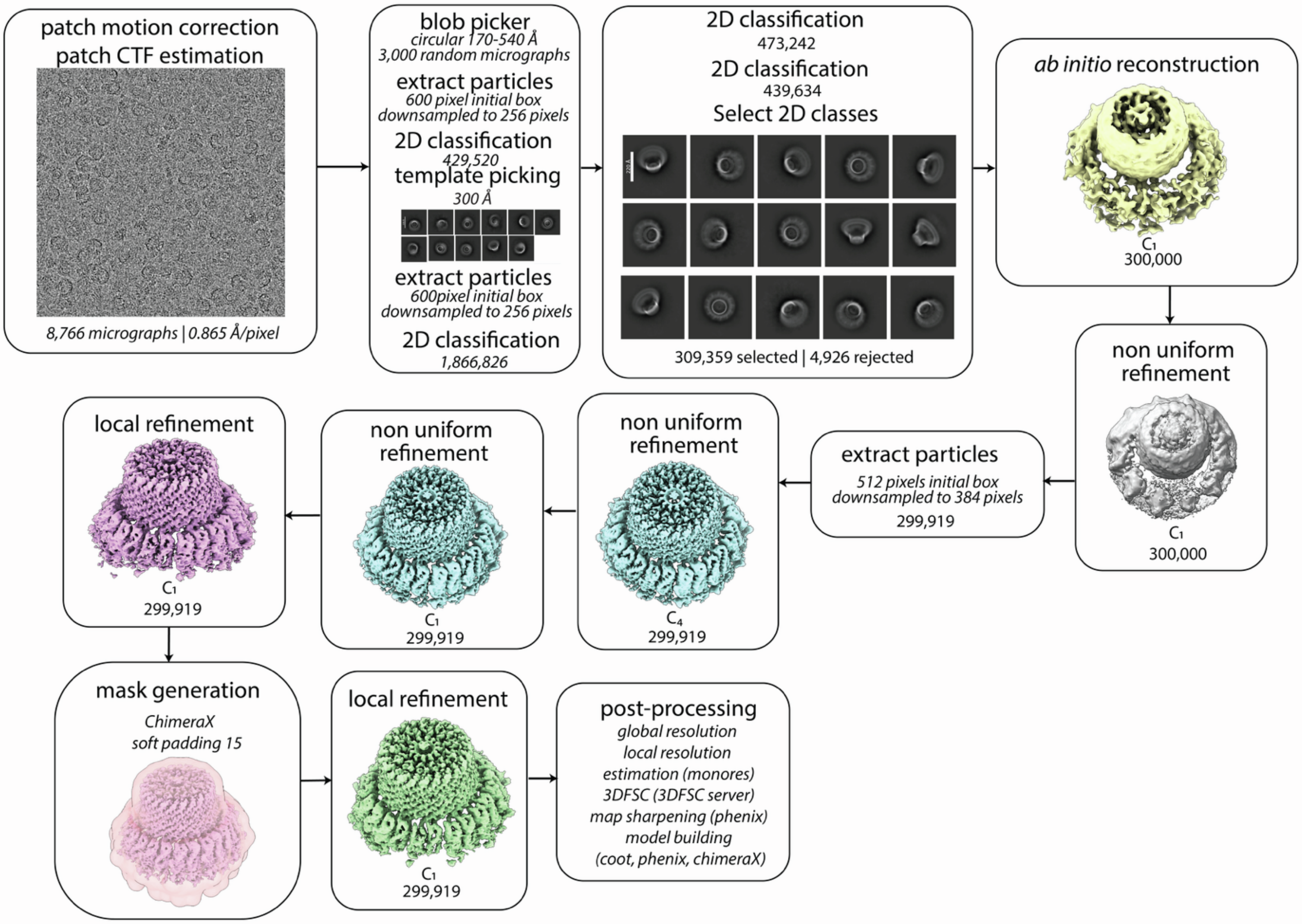

Supplementary Figure 1. CryoSPARC processing workflow for cross-linked HflK/C^{SS} complex. Job names, job details, and non-default parameters (italicized) are noted in each box.

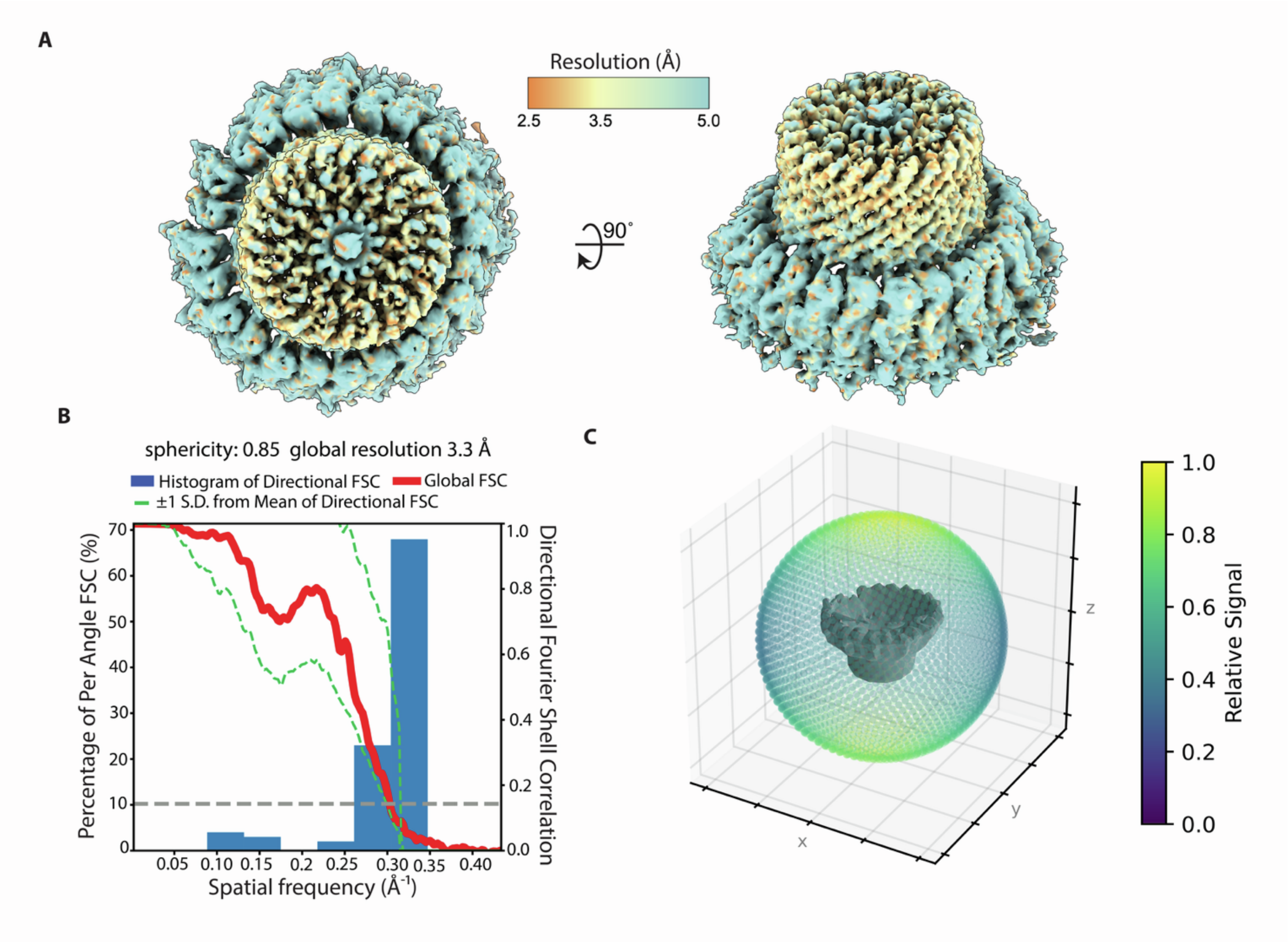

Supplementary Figure 2. Estimates of resolution and angular sampling: DDM-solubilized FtsH•HflK/C^{SS} complex. (A) Maps colored by local resolution as estimated by the cryoSPARC implementation of monoRes. **(B)** Global resolution and directional resolution calculated by 3DFSC server (https://3dfsc.salk.edu). **(C)** Projection angle distribution estimated by cryoSPARC.

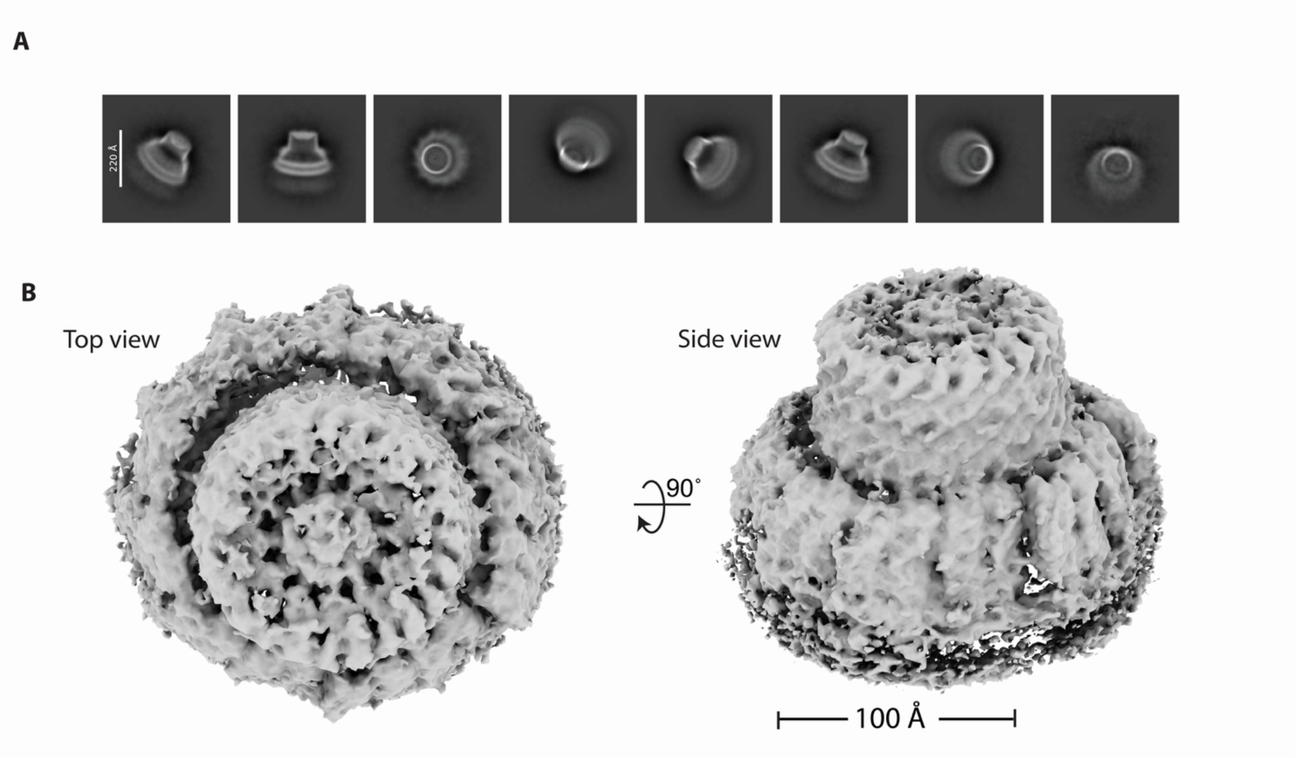

Supplementary Figure 3. Cryo-EM structure of the crosslinked, GDN-solubilized FtsH–HflK/C (HflK/C^{SS}) complex and low-pass-filtered map of the DDM-solubilized HflK/C^{SS} complex. (**A**) Representative 2D class averages of the crosslinked complex. (**B**) Cryo-EM map of the final 3D reconstruction, resolved at global GS-FSC resolution: 4.14 Å.

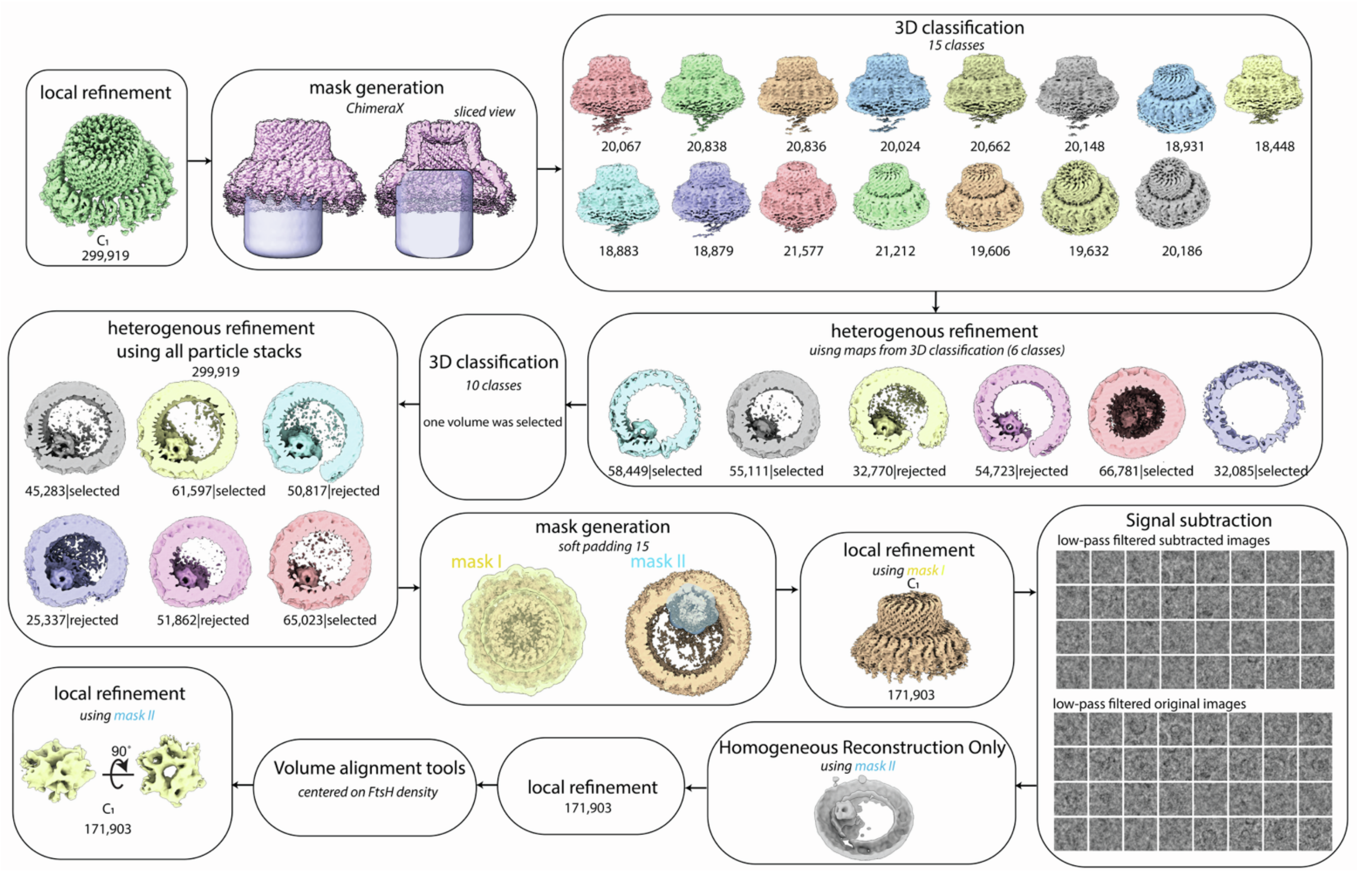

Supplementary Figure 4. Heterogeneity analysis of DDM-solubilized FtsH•HflK/C^{SS} confirm the presence of FtsH within the assembly. Job names, job details, and non-default parameters (italicized) are noted in each box.

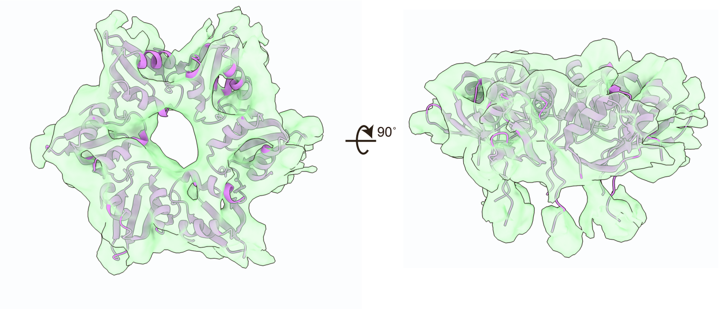

Supplementary Figure 5. Visualization of the FtsH periplasmic region within the cross-linked HflK/C (HflK/C^{SS}). (**B**) The FtsH periplasmic region obtained from signal subtraction, overlaid with the model of the FtsH periplasmic region from PDB ID: 9CZ2.

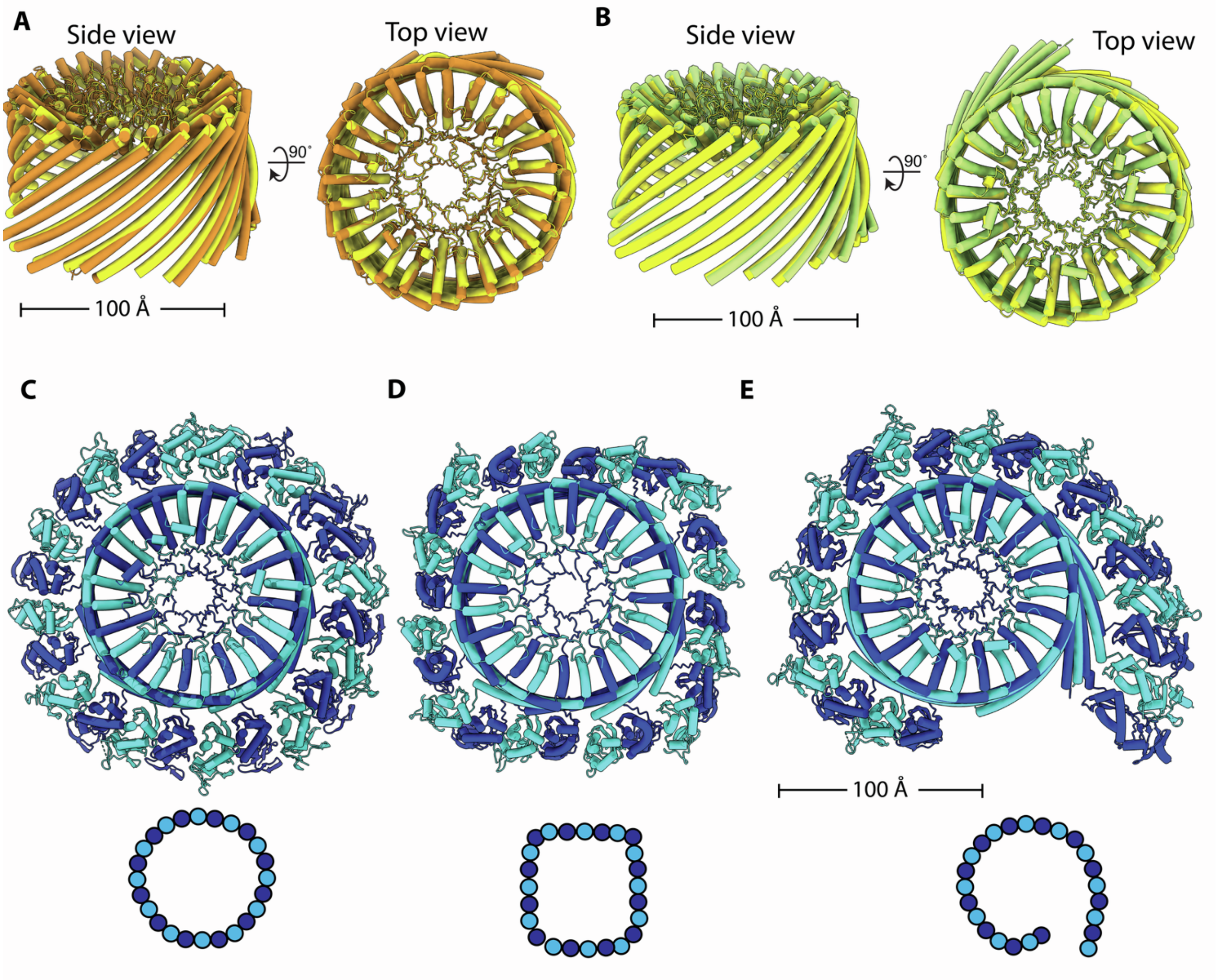

Supplementary Figure 6. Comparison of the cross-linked HflK/C^{SS} closed conformation with previously determined closed and open structures. (**A**) Overlay of the "hat" region from HflK/C^{SS} (yellow) and the previously reported closed 'cage' structure (PDB ID: 7WI3, orange). The closed 'cage' structure was determined using both overexpression and glutaraldehyde crosslinking. (**B**) Overlay of the "hat" region from HflK/C^{SS} (yellow) and the open, nautilus-like structure (PDB ID: 9CZ2, green). Only the HflK and HflC subunits located at the opening in the nautilus-like conformation exhibit conformational differences compared to the cross-linked HflK/C^{SS} structure. (**C–E**) Top-view comparisons of (**C**) the fully closed HflK/C^{SS} structure (**D**) the previously reported closed conformation (PDB ID: 7WI3); and (**E**) the open, nautilus-like open conformation of the native HflK/C complex (PDB ID: 9CZ2). All panels show cartoon representations with HflK in blue and HflC in cyan. The bottom panels show the curvature of the assemblies.

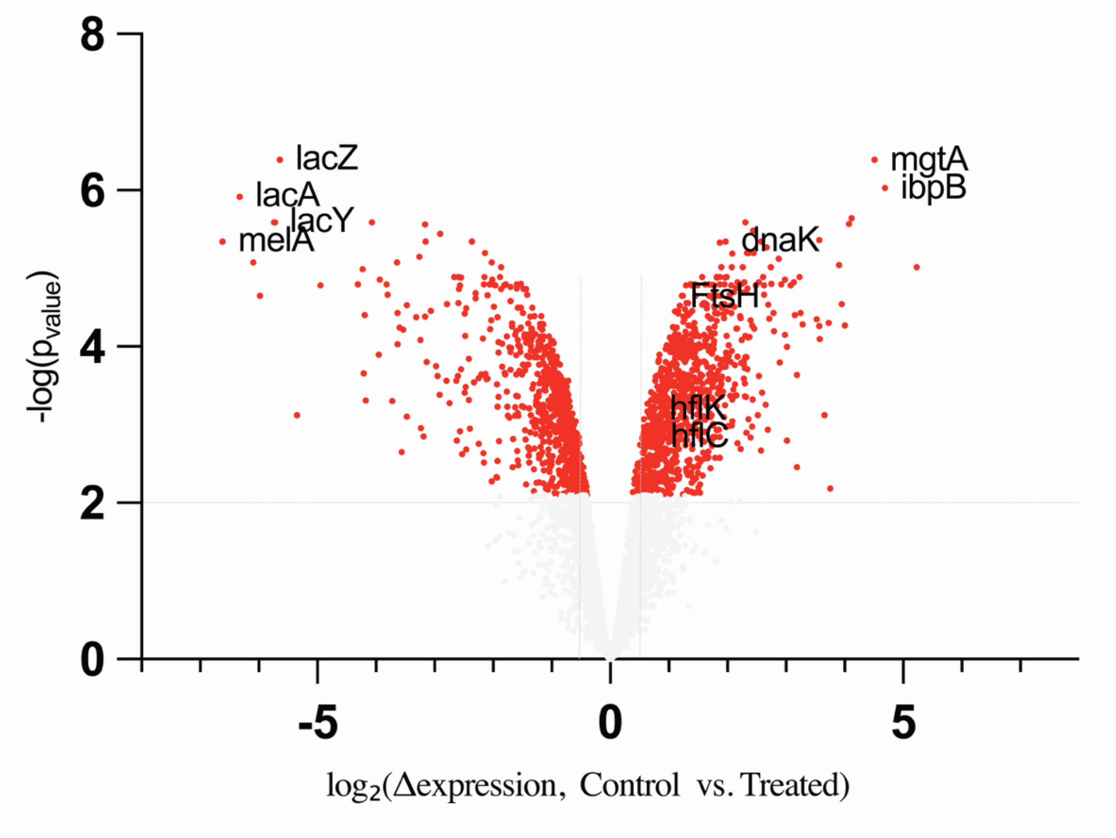

Supplementary Figure 7. Volcano plot of RNA-seq data comparing control and tobramycin-treated *E. coli* BL21 cells. Each point represents a gene, plotted by $\log_2$ fold change (x-axis) and statistical significance (−log*P*-value, *y*-axis). Genes with $-\log P > 2$ and $\log_2 \Delta\text{expression} > 0.5$ are considered significantly upregulated (red). Notably upregulated genes include *ftsH*, *hflK*, *hflC*, *mgtA*, *ibpB*, and *dnaK*, a chaperone known to interact with FtsH. Downregulated genes include *lacZ*, *lacA*, *lacY*, and *melA*.

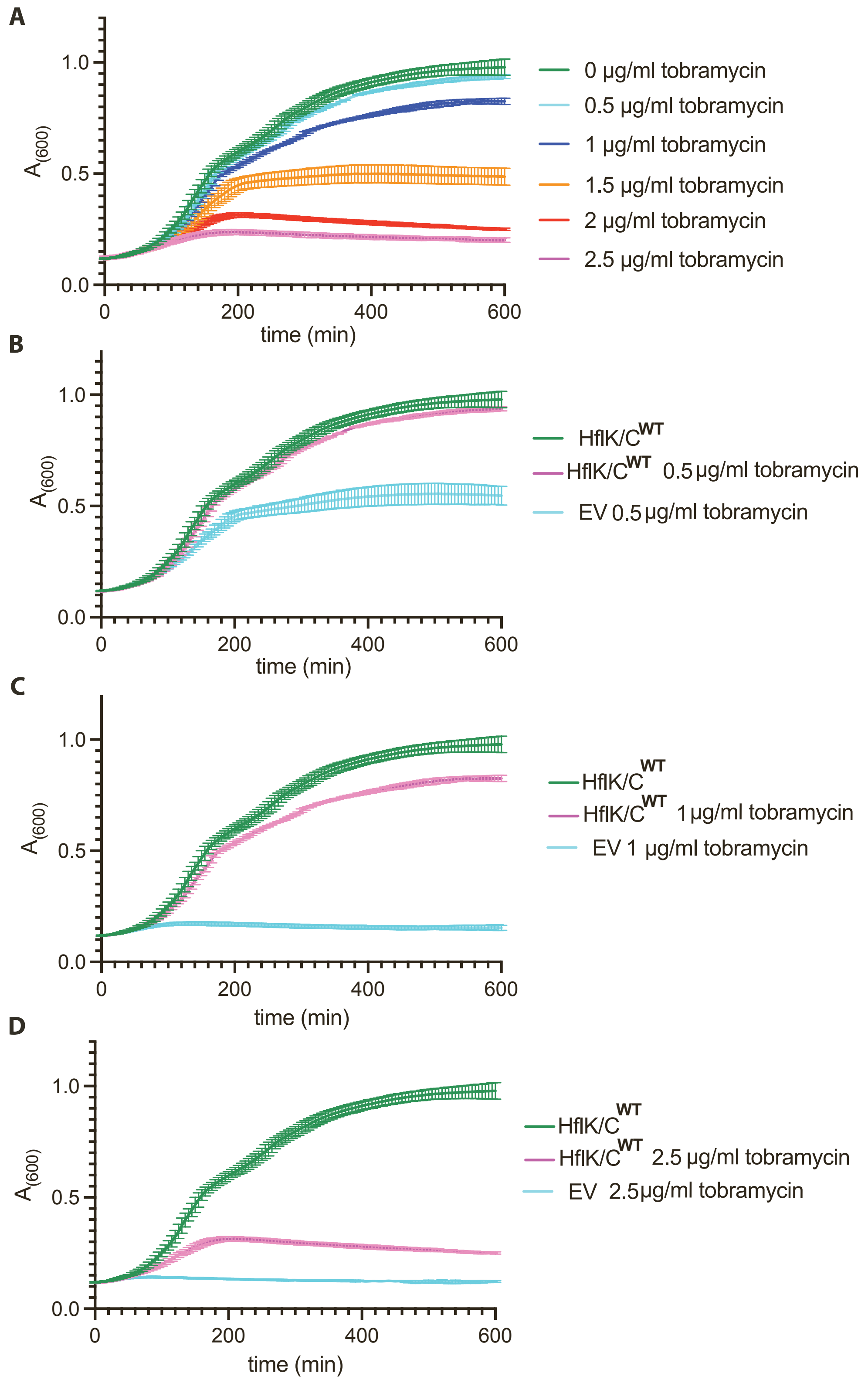

Supplementary Figure 8. Inhibitory concentration of tobramycin for growth assays. Growth assays were conducted using *E. coli* BL21 Δ*hflK/C* cells expressing wild-type HflK/C (HflK/C^{WT}) or an empty vector from a pPro24 plasmid under the control of a sodium propionate-inducible promoter at 37°C in LB medium. (**A**) Growth of cells expressing HflK/C^{WT} across a range of tobramycin concentrations. (**B–D**) Growth rate comparisons between HflK/C^{WT} and empty vector-expressing cells at 0.5, 1, and 2.5 µg/mL tobramycin, respectively. Each growth assay was performed from a separate colony (n = 3 independent biological replicates), and data are presented as mean ± 1 SD.

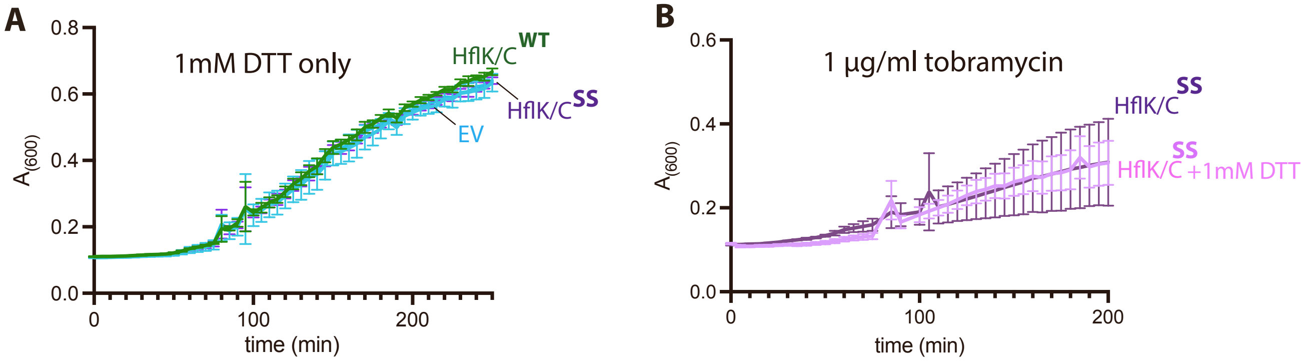

Supplementary Figure 9. Testing whether DTT addition can restore growth in cells expressing cross-linked HflK/C *in vivo* under tobramycin stress. Growth assays were conducted using *E. coli* BL21 Δ*hflK/C* cells expressing wild-type HflK/C (HflK/C^{WT}), cross-linked HflK/C ((HflK/C^{SS}), or an empty vector from a pPro24 plasmid under the control of a sodium propionate-inducible promoter at 37°C in LB medium in the presence of 1mM sodium propionate. (**A**) Growth of cells under 1 mM DTT only. (**B**) Growth rate comparisons between cells expressing HflK/C^{SS} in the presence or absence of DTT under 1µg/ml tobramycin stress. Each growth assay was performed from a separate colony (n = 3 independent biological replicates), and data are presented as mean ± 1 SD.

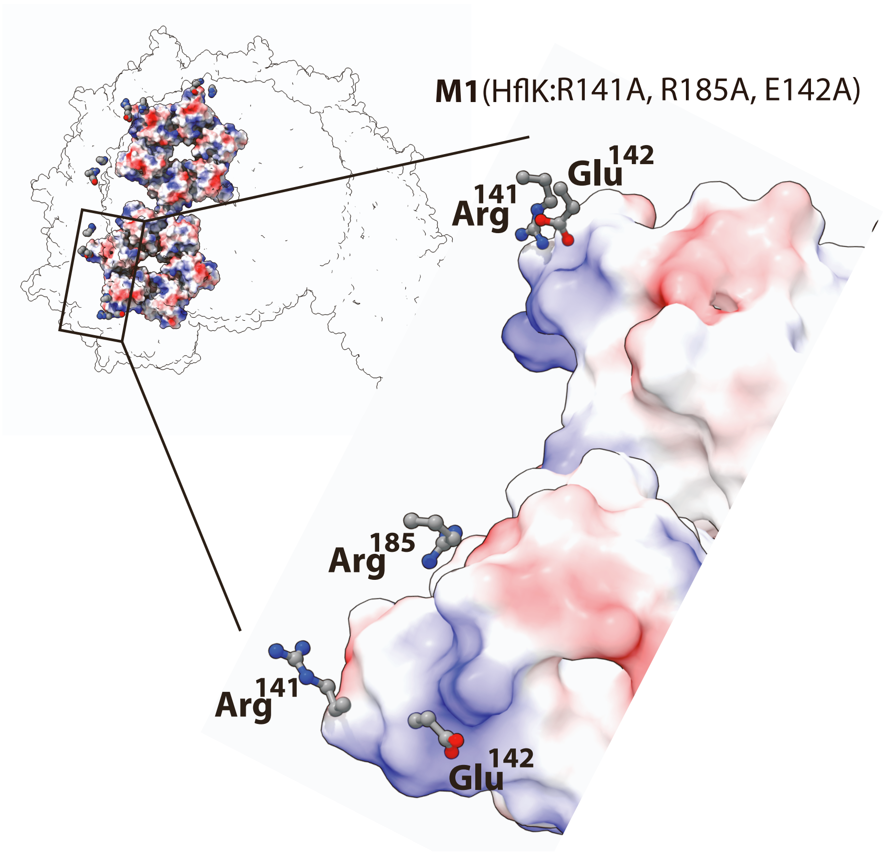

Supplementary Figure10. Mutational analysis of interface residues in the M1 HflK variant (R141A, E142A, R185A) within the HflK/C–FtsH complex. Interface residues were identified based on the structural model of the FtsH•HflK/C assembly (PDB: 9CZ2).

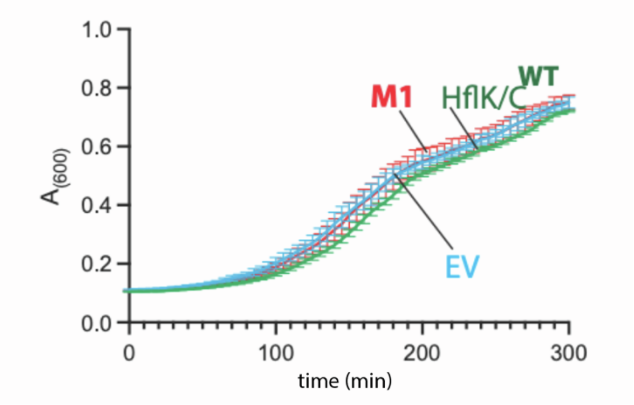

Supplementary Figure 11. Growth assays for M1 mutants under a standard growth condition. Growth assays were performed using *E. coli* BL21 Δ*hflK/C* cells expressing wild-type HflK/C(HflK/C^{WT}), **M1**, or an empty vector from a pPro24 plasmid under the control of a sodium propionate-inducible promoter at 37°C in LB medium. The growth assay was performed from a separate colony (n = 3 independent biological replicates), and data are presented as mean ± 1 SD.

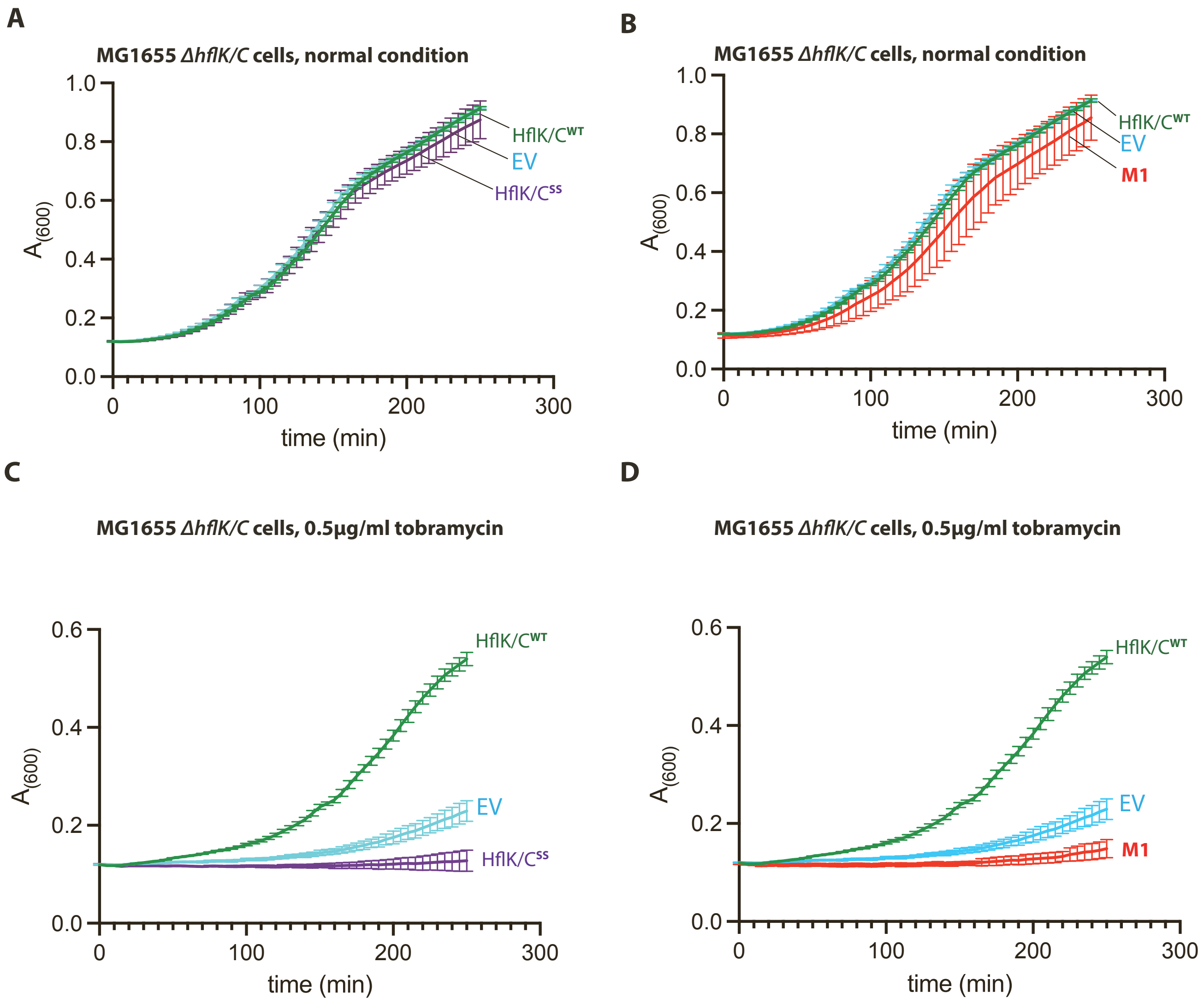

Supplementary Figure 12. Effects of HflK/C variants on bacterial recovery under aminoglycoside stress using MG1655 cells. Growth assays were conducted using *E. coli* MG1655 Δ*hflK/C* cells expressing wild-type HflK/C (HflK/C^{WT}), crosslinked HflK/C (HflK/C^{SS}), **M1,** or an empty vector from a pPro24 plasmid at 37 °C in LB medium containing 35 µg/ml chloramphenicol and 1 mM sodium propionate unless otherwise noted. (**A**) Growth curves for cells expressing wild-type HflK/C (HflK/C^{WT}), crosslinked HflK/C (HflK/C^{SS}), and empty vector (EV). (**B**) Growth curves for cells expressing HflK/C^{WT}, **M1** (R141A, E142A, R185A), and EV. (**C**) Growth curves for cells expressing HflK/C^{WT}, HflK/C^{SS}, and EV under tobramycin (0.5µg/ ml) stress.(**D**) Growth curves for cells expressing HflK/C^{WT}, **M1**, and EV under tobramycin (0.5µg/ ml) stress. Each growth assay was performed from a separate colony (n = 3 independent biological replicates), and data are presented as mean ± 1 SD.

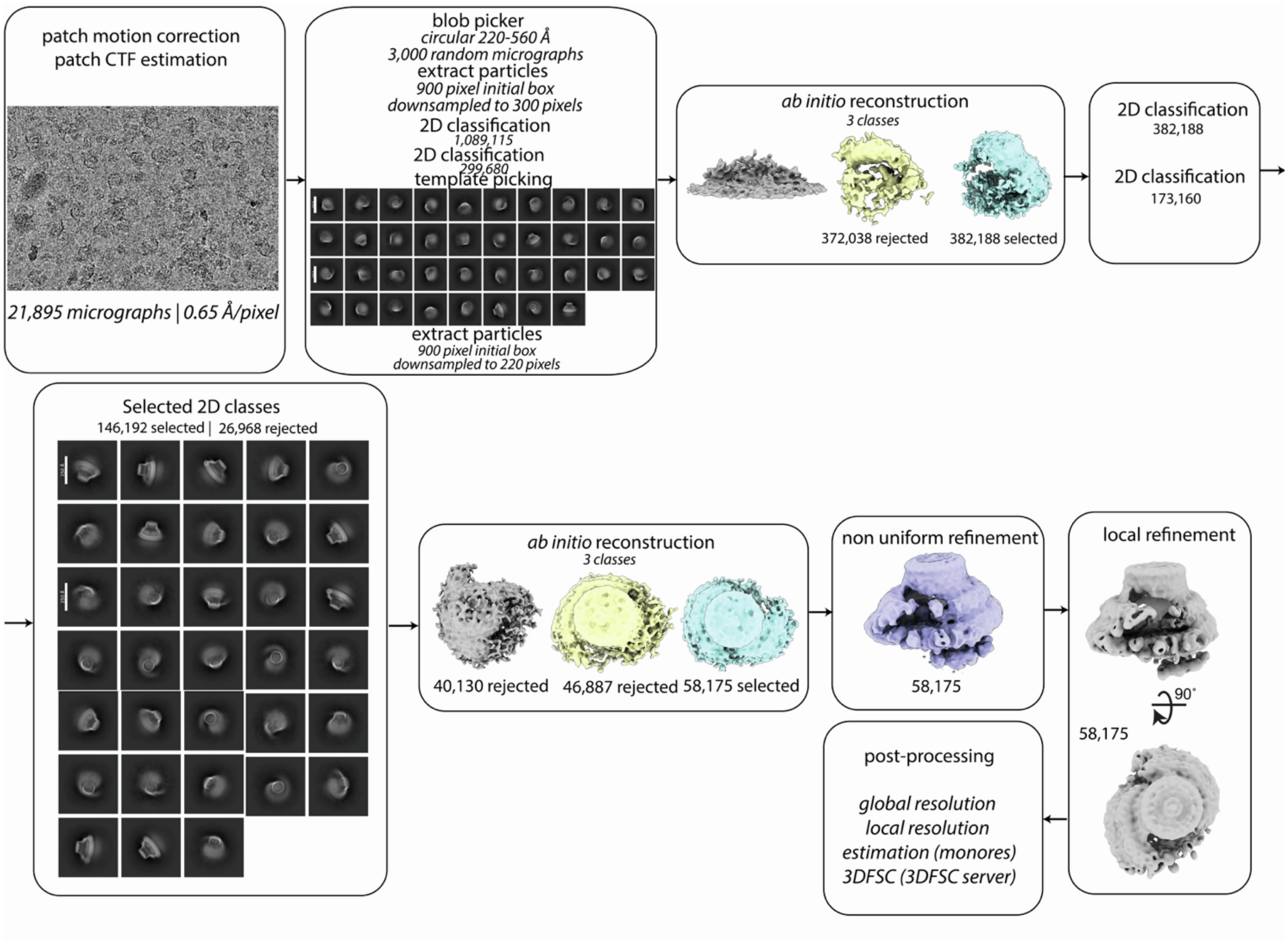

Supplementary Figure 13. CryoSPARC processing workflow for the FtsH•HflK/C complex solubilized in DDM from cells treated with the aminoglycoside tobramycin. The workflow outlines the cryo-EM data processing steps, including job names, job details, and any non-default parameters (italicized).

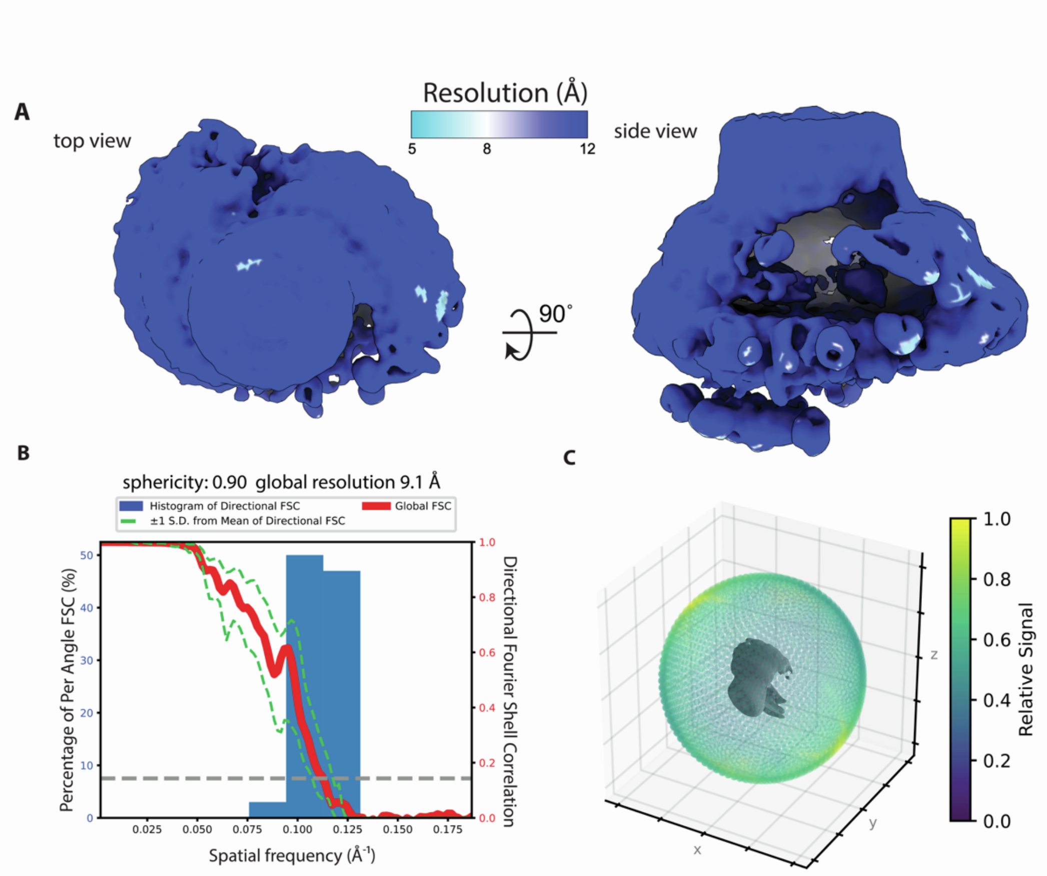

Supplementary Figure 14. Estimates of resolution and angular sampling: DDM-solubilized FtsH•HflK/C complex extracted from the tobramycin treated cells. **(A)** Maps colored by local resolution as estimated by the cryoSPARC implementation of monoRes. **(B)** Global resolution and directional resolution calculated by 3DFSC server (https://3dfsc.salk.edu). **(C)** Projection angle distribution estimated by cryoSPARC.

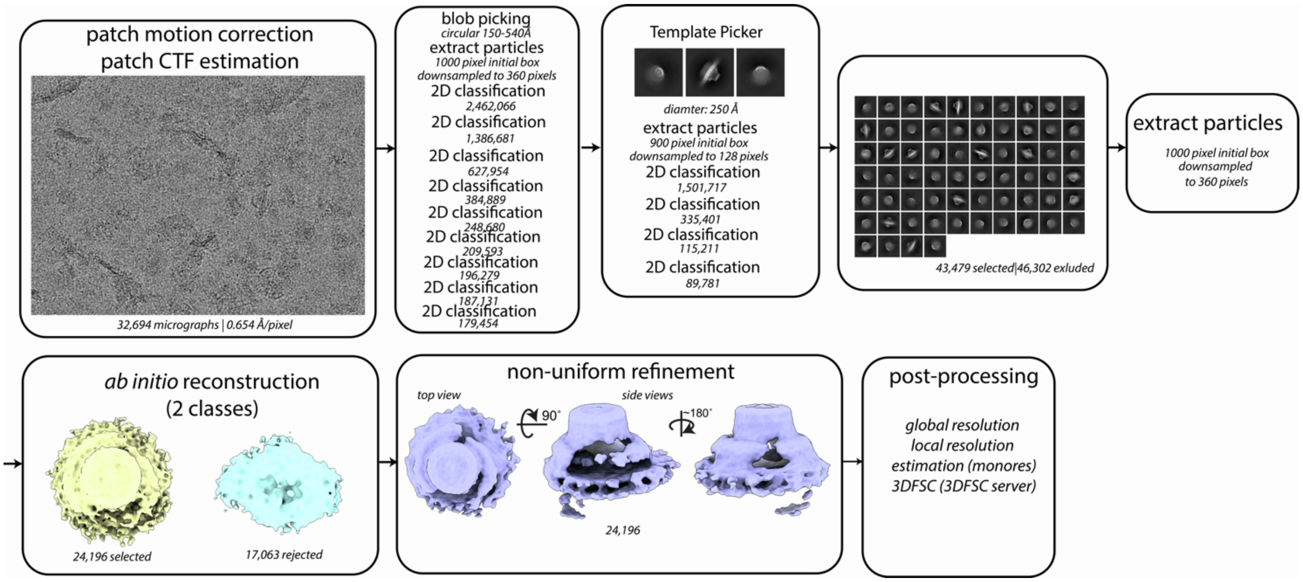

Supplementary Figure 15. CryoSPARC processing workflow for the FtsH•HflK/C complex reconstituted in Carboxy-DIBMA from cells treated with the aminoglycoside tobramycin. The workflow outlines the cryo-EM data processing steps, including job names, job details, and any non-default parameters (italicized).

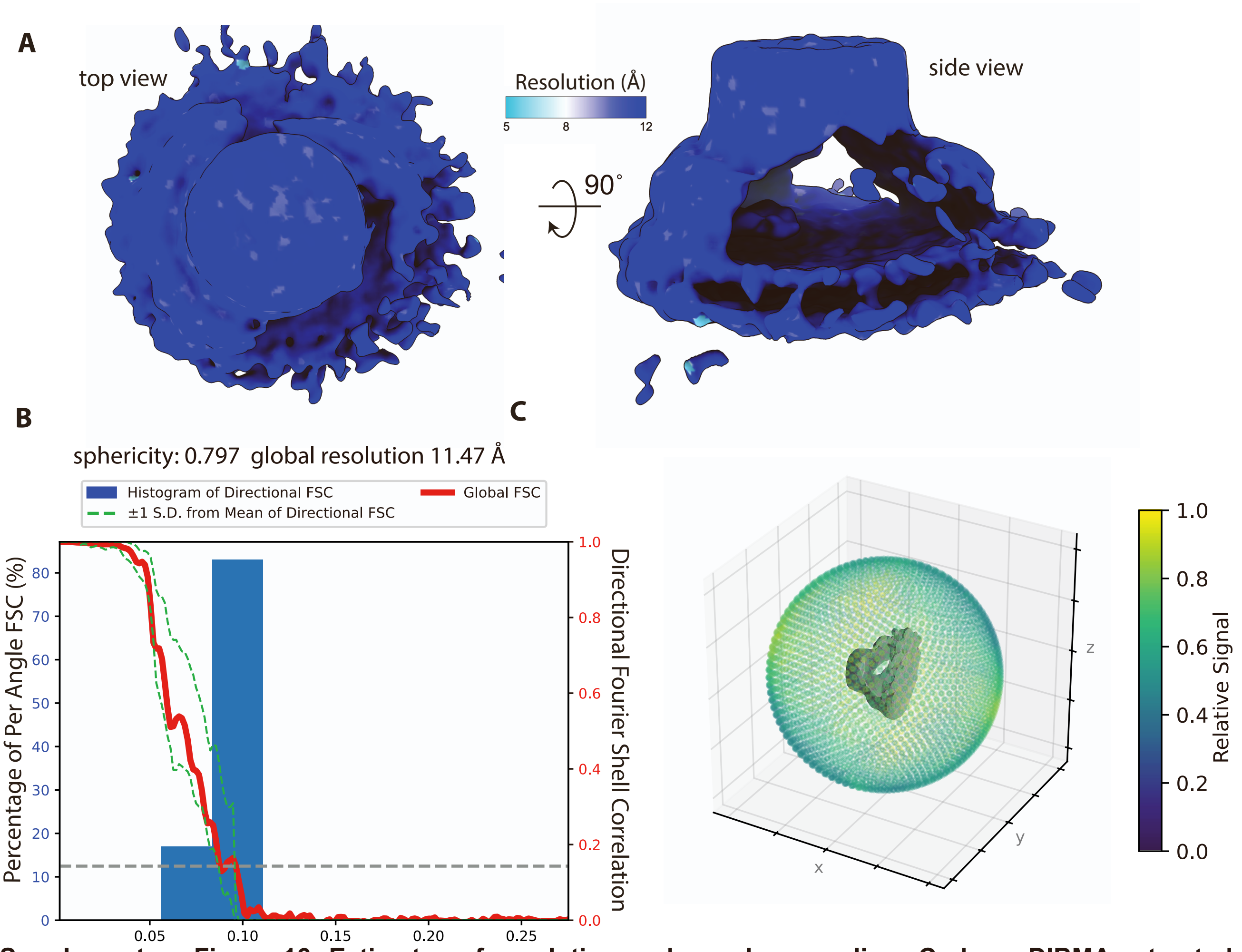

Supplementary Figure 16. Estimates of resolution and angular sampling: Carboxy-DIBMA-extracted FtsH•HflK/C complex obtained from tobramycin-treated cells (map a). **(A)** Maps colored by local resolution as estimated by the cryoSPARC implementation of monoRes. **(B)** Global resolution and directional resolution calculated by 3DFSC server (https://3dfsc.salk.edu). **(C)** Projection angle distribution estimated by cryoSPARC.
